# Antioxidant Properties of Camphene-Based Thiosemicarbazones: Experimental and Theoretical Evaluation

**DOI:** 10.3390/molecules25051192

**Published:** 2020-03-06

**Authors:** Lijuan Yang, Haochuang Liu, Dasha Xia, Shifa Wang

**Affiliations:** 1 College of Chemical Engineering, Nanjing Forestry University, Nanjing 210037, China; lijuan_yang@163.com (L.Y.); lhchuang96@163.com (H.L.); 2 Hangzhou Yanqu Information Technology Co., Ltd., Hangzhou 310012, China; xiadasha@hotmail.com; 3 Co-Innovation Center of Efficient Processing and Utilization of Forest Resources, College of Chemical Engineering, Nanjing Forestry University, Nanjing 210037, China

**Keywords:** camphene-based thiosemicarbazones, DPPH, peroxyl radical scavenging capacity, DFT

## Abstract

The thiosemicarbazone derivatives have a wide range of biological activities, such as antioxidant activity. In this study, the antiradical activities of six camphene-based thiosemicarbazones (**TSC-1~6**) were investigated by 2,2-diphenyl-1-picrylhydrazyl (DPPH) and peroxyl radical scavenging capacity (PSC) assays, respectively, and the results reveal that **TSC1~6** exhibited good abilities for scavenging free radicals in a dose-dependent way. Compound **TSC-2** exhibited the best effect of scavenging DPPH radical, with the lowest EC_50_ (0.208 ± 0.004 mol/mol DPPH) as well as the highest bimolecular rate constant K_b_ (4218 M^−1^ s^−1^), which is 1.18-fold higher than that of Trolox. Meanwhile, **TSC-2** also obtained the lowest EC_50_ (1.27 µmol of Trolox equiv/µmol) of scavenging peroxyl radical. Furthermore, the density functional theory (DFT) calculation was carried out to further explain the experimental results by calculating several molecular descriptors associated with radical scavenging activity. These theoretical data suggested that the electron-donating effect of the diethylamino group in **TSC-2** leads to the enhancement of the scavenging activities and the studied compounds may prefer to undergo the hydrogen atom transfer process.

## 1. Introduction

Oxidation is an essential part of aerobic metabolism in living organisms. In the complicated oxidation process, free radicals are constantly generated containing reactive oxygen species (ROS) and reactive nitrogen species (RNS) [1,2]. It is widely known that free radicals play a dual role in vivo as both beneficial and deleterious compounds [3,4,5]. The beneficial effects of free radicals involved important physiological roles in the function of a number of intercellular signaling pathways etc. [5,6]. In contrast, excessive free radicals can cause irreversible tissue injury by attacking biomolecules such as lipids, proteins, and DNA [7,8]. Therefore, this balance is essential for the survival of organisms and their health [3]. In order to maintain the balance of the activity of the free radicals in living cells, the organism has evolved antioxidant defense systems, protecting against free radical damage [9,10]. However, when our endogenous defense system has incomplete efficiency, or a rise in free radicals under special conditions (smoking, chemical air pollutants, radiation, and inflammation), the imbalance between free radicals and antioxidants results in oxidative stress (oxidative damage), which is now believed to be one of the important factors in the occurrence of certain human diseases including atherosclerosis, rheumatoid arthritis, cancer, and neurodegenerative diseases [11,12]. So, antioxidants have aroused the interest of biomedical scientists and clinicians because they protect the body against damage by harmful free radicals [13]. In addition, it is necessary to explore some effective radical scavengers.

Thiosemicarbazone is a well-known class of ligand with a broad spectrum of biological activities, including antibacterial, antifungal, antiviral, antimalarial, anticancer, and antioxidant activities [14,15,16,17,18,19,20,21]. Therefore, thiosemicarbazone has attracted increasing research attention in the pharmaceutical industry. As a bicyclic monoterpene, camphene has been broadly employed as a starting material in the synthesis of biomolecules for its low cost and broad availability [22]. Moreover, camphene was reported as having high hypolipidemic action [23] and antioxidant activity [22]. In the earlier reports, the synthesis and potential activity of camphene-based thiosemicarbazone derivatives have been reported [24,25]. For example, Mariana reported camphene-based thiosemicarbazone compounds with anti-tuberculosis activity [26]. A series of camphene-based thiosemicarbazone derivatives were synthesized and their antitumor activity was evaluated by our research group [27]. Nevertheless, there are few reports on the antioxidant activities of camphene-based thiosemicarbazones. 

Considering the above-mentioned reasons and as an extension of our previous work, in this work we attempted to experimentally and theoretically evaluate the antioxidant behavior of six camphene-based thiosemicarbazone compounds (**TSC-1~6**), which were selected from a series of previously synthesized compounds [27]. The experimental results show that the compound 2-hydroxy-4-(N, N-diethylamino) benzaldehyde-4-(2′-isocamphanyl) thiosemicarbazone **(TSC-2)** possesses high scavenging radical activity. Furthermore, the DFT approach was employed to elucidate scavenging free radical mechanisms theoretically [28,29,30]. This study will assist in the design of thiosemicarbazone derivatives with the desired antioxidant properties.

## 2. Results and Discussion

**Figure 1:**
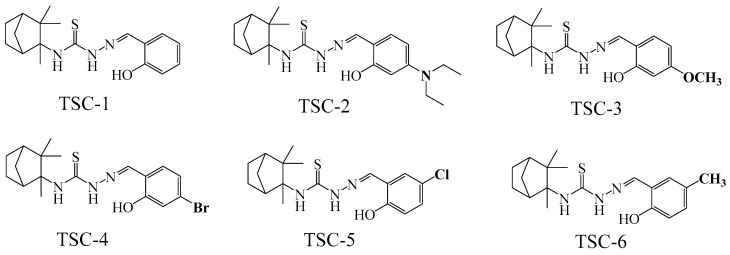
Molecular structures of the studied compounds **TSC-1~6**.

The six camphene-based thiosemicarbazone (**TSC1-6**) are depicted in Figure 1.

### 2.1. Radical Scavenging Activity

#### 2.1.1. 2,2-diphenyl-1-picrylhydrazyl (DPPH) Radical Scavenging Assay

**Figure 2:**
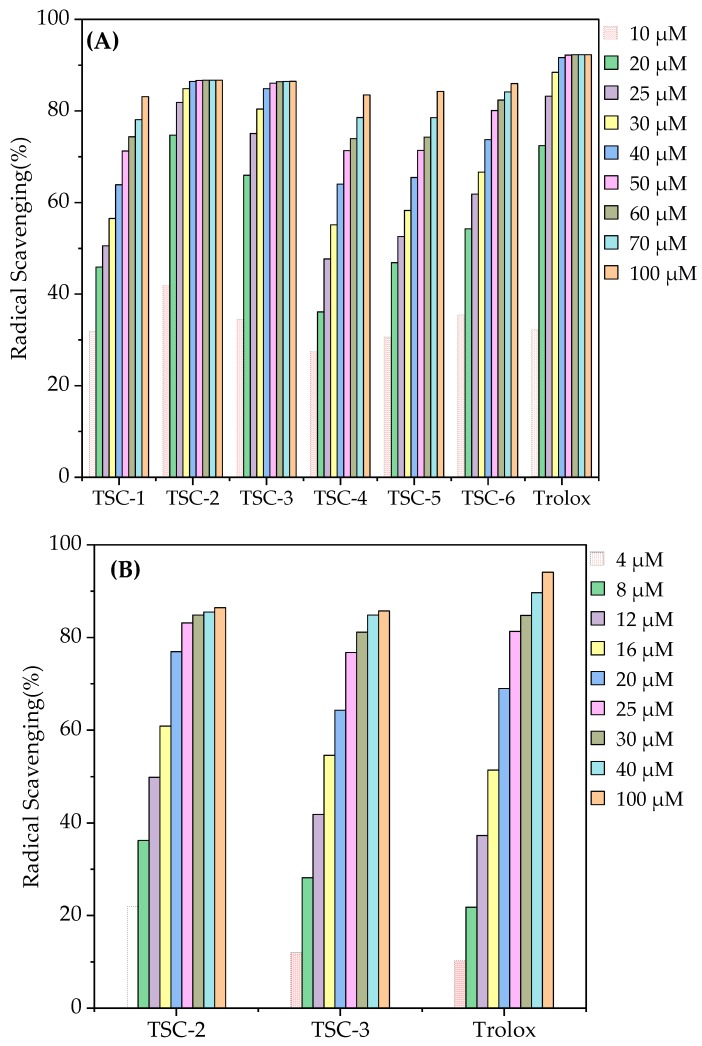
(**A**) 2,2-diphenyl-1-picrylhydrazyl (DPPH) radical scavenging capacity (%) of the camphene-based thiosemicarbazone compounds (**TSC-1~6**) at different concentrations (10~100 µM); (**B**) DPPH radical scavenging capacity (%) of compounds **TSC-2**, **TSC-3**, and Trolox at different concentrations (4~100 µM).

DPPH radical is a stable radical, and has an odd electron with a strong absorption band at 517 nm in ethanol solution. When the DPPH radical is reduced by acquiring an electron or hydrogen atom from the antioxidant, the color of DPPH alcohol solution turns from deep violet to pale yellow; meanwhile, the intensity of the absorption peak at 517 nm decreases [31]. As shown in Figure 2A, all of the compounds displayed appreciable DPPH radical scavenging activity, compared with Trolox. In the range of 10–100 µM, with the increasing concentration, the DPPH scavenging rate of **TSC-1**, **TSC-4**, **TSC-5**, and **TSC-6** showed considerable dose-dependent activity and reached the maximum of about 83% at 100 µM. For **TSC-2** and **TSC-3**, they showed higher scavenging activity at 25 µM, while with a further increase in concentration the scavenging rate did not show a significant change. Figure 2B shows that in the range of 4–25 µM, **TSC-2** and **TSC-3** showed dose-dependent activity. Among these compounds, **TSC-2** and **TSC-3** had more remarkable scavenging DPPH activity than others.

The non-linear regression model [32] was applied to fit the experimental data to obtain the EC_50_ (Table 1). As showed in Table 1, **TSC-2** exhibited the highest DPPH scavenging activity, which is higher than that of Trolox. The EC_50_ value of **TSC-3** was almost the same compared with Trolox. The EC_50_ values of **TSC-1**, **TSC-4**, **TSC-5**, **TSC-6** were higher than the reference.

**Figure 3:**
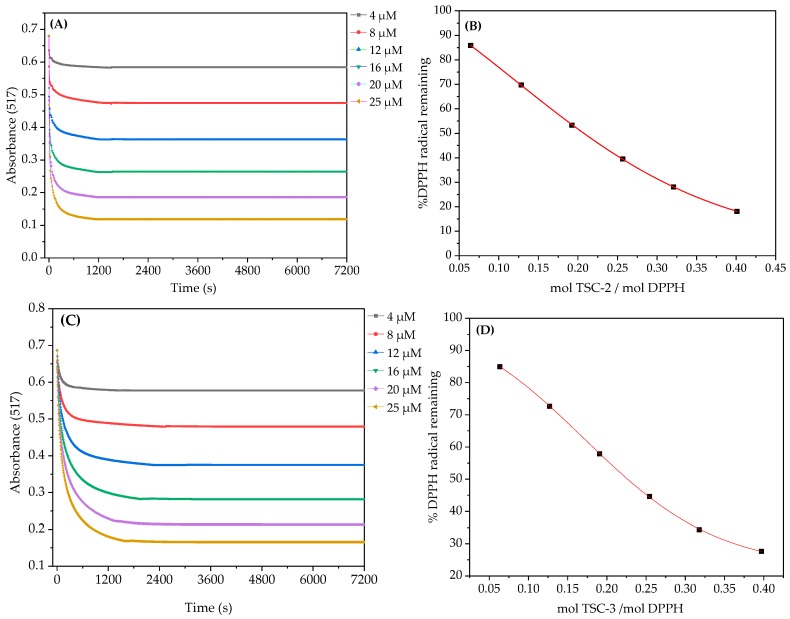
Reaction kinetics of DPPH radical with **TSC-2** (**A**) and **TSC-3** (**C**); %DPPH radical remaining at infinite time at the different concentration of **TSC-2** (**B**)and **TSC-3** (**D**).

The reaction of DPPH radical with antioxidants is basically a kinetic driving process [33]. So, it is necessary to assess the kinetic behavior between DPPH radical and antioxidants. Two processes occurred, containing a rapid process in the first few seconds followed by a slower one at longer reaction times. According to the literature [34], it was detected that the reaction was best modeled by an empirical bi-exponential decay function. (Figure 3 and Figure S1).

**Figure 4:**
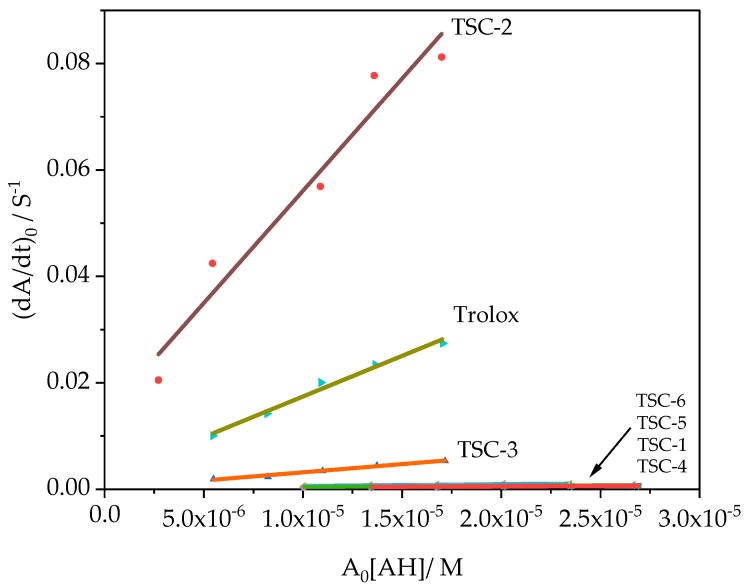
Linear plots of (dA/dt)_0_ vs. A_0_[AH] of compounds. A_0_ is the absorbance at time = 0, and [AH] is the concentration of antioxidant at time = 0, R^2^ > 0.9500.

By analyzing the data as described in the experimental portion, the EC_50_ (mol AH/mol DPPH), stoichiometric factor(*n*) and the bimolecular rate constants(k_b_) were procured (Table 1), which are important parameters to consider when investigating antioxidants. Trolox was used as a reference in the DPPH kinetic assay. Regarding stoichiometric factor (n), the four tested compounds **TSC-1**, **TSC-4**, **TSC-5**, and **TSC-6** have stoichiometric factors ranging from 1.21 to 1.44. The Trolox has a stoichiometric factor of 1.86. **TSC-2** and **TSC-3** had higher stoichiometric factor values of almost the same value—2.27. As illustrated in Figure 4, the ${\left(dA/dt\right)}_{0}$ increased linearly with A_0_[AH]. From the slope of a linear plot of ${\left(dA/dt\right)}_{0}$ against A_0_[AH], the bimolecular rate constants (k_b_) for the radical scavenging reaction were determined and presented in Table 1. 

There were obvious differences in reactivity between the tested compounds. In the combined reaction kinetics (Figure 3 and Figure S1), it can be found that **TSC-2** reacted very quickly with DPPH with the largest bimolecular rate constant K_b_ value (4218 M^−1^S^−1^), larger than the k_b_ of Trolox (3570 M^−1^S^−1^). The K_b_ value of **TSC-2** is 1.18-fold higher than Trolox, while the K_b_ value of **TSC-3** is one magnitude less than Trolox. Based on k_b_ value, the sort of scavenging reactivity was **TSC-2** > Trolox > **TSC-3** >> **TSC-6** > **TSC-5** > **TSC-1** > **TSC-4**. The results suggest that compounds with higher stoichiometric factor values tend to have higher K_b_ values.

Combined with the above results, it could be found that the substituents on the phenyl ring have a significant effect on the scavenging activity. As observed, the electron-donating groups (diethylamino group, methoxy group) can dramatically enhanced radical-scavenging activity, which explains why **TSC-2** possessed the highest activity with the most electron-donating diethylamino group attached on the phenyl ring.

#### 2.1.2. Peroxyl Radical Scavenging Capacity Assay

Peroxyl radical scavenging capacity assay is a simple, rapid and sensitive assay for evaluating the antioxidant activity of both hydrophilic and lipophilic antioxidants. The reaction mechanism of this essay has been described in the previous literature [35]. Briefly, the thermal degradation of 2,2′-azobis(2-amidinopropane) dihydrochloride (AAPH) produces peroxyl radicals (ROO^•·^) which oxidize non-fluorescent dichlorofluorescein (DCFH) to fluorescent dichlorofluorescein (DCF). The degree of inhibition of DCFH oxidation by antioxidants was used for evaluating the antioxidant activity [36].

**Figure 5:**
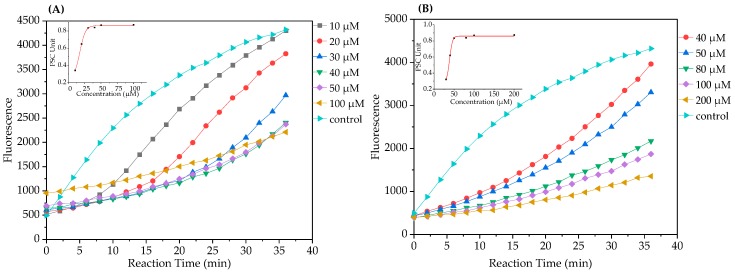
Time kinetics plots of **TSC-2** (**A**) and **TSC-3** (**B**) inhibition of non-fluorescent dichlorofluorescein (DCFH) oxidation by 2,2′-azobis(2-amidinopropane) dihydrochloride (AAPH). (the inset is dose-response plots).

The peroxyl radical scavenging capacity assays of six tested compounds and Trolox are determined in Figure 5 and Figure S2. 

It can be seen that ROO^•^ oxidizes DCFH to fluorescent DCF over time and antioxidants scavenged peroxyl radicals and inhibited the oxidation reaction in a dose-dependent way. Scavenging peroxyl radical activity was expressed as EC_50_ and as micromoles of Trolox equivalents per micromole of the test sample. The degree of inhibition was different for the investigated compounds, as shown in Table 1. Among the six compounds, **TSC-2** had the highest PSC value and that indicated that **TSC-2** possess the highest peroxyl radical-scavenging activity, which was 1.27 times more than Trolox. Meanwhile, the peroxyl radical scavenging capacities of the other compounds were lower than Trolox. The order of antioxidant activity for tested compounds was **TSC-2 > Trolox > TSC-3 > TSC-6 > TSC-4 > TSC-5 > TSC-1**, which was similar to the result of the DPPH assay.

### 2.2. Theoretical Calculations

During the past few decades, theoretical calculations based on DFT have become a potent method to elucidate the antioxidant properties of compounds [28,29,37,38]. In order to further understand the scavenging activity of the tested compounds, the theoretical calculation was performed at DFT level through the Gaussian 09 software package [39]. The geometry optimizations and frequency calculations of the investigated species were carried out using the M06-2X/Def2-SVP level and B3LYP/6-31G(d) level of theory, in conjunction with the polarizable continuum solvation (PCM) model [40], using ethanol as the solvent.

**Figure 6:**
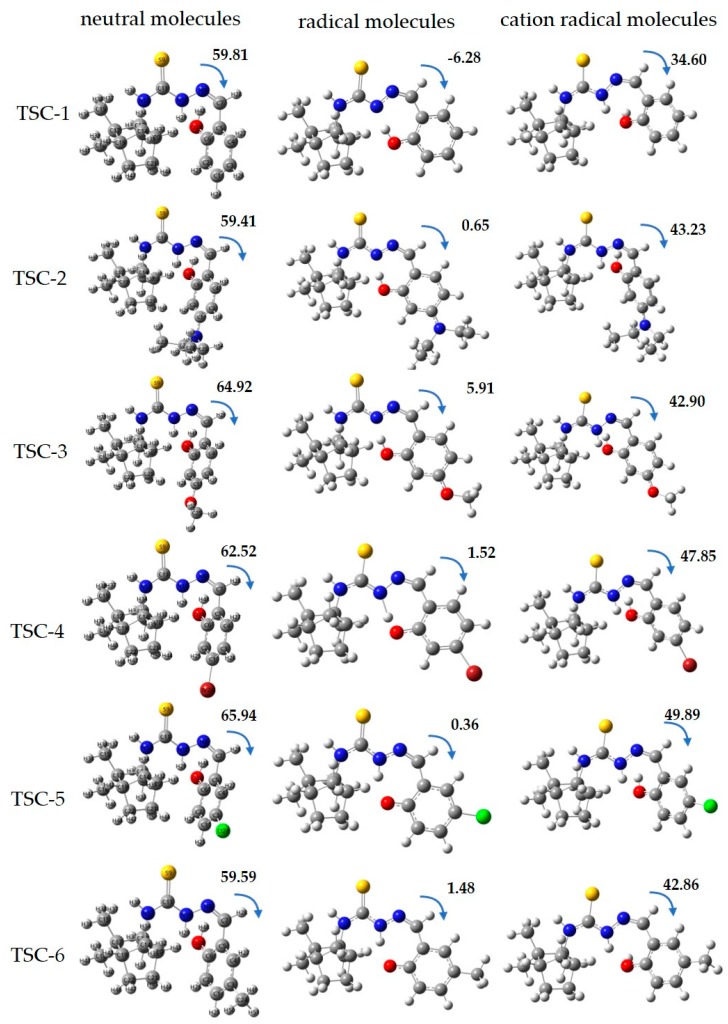
Geometric structure of the neutral molecules, radical molecules, cation radical molecules and atomic number of the studied camphene-based thiosemicarbazone. The blue, gray, yellow, white and red balls represent N atom, .C atom, S atom, H atom and O atom respectively.

First at all, the conformational analysis was first performed to identify the most stable conformational structures of the studied compounds. The optimized structures of the most stable compounds were shown in Figure 6. The main geometrical parameters of the studied compounds including bond length and dihedral angle are listed in Table S1 in supporting information, where the cartesian coordinates of compounds are available.

As shown in Figure 6, the camphene-based thiosemicarbazone structures consisted of a camphene-based thiosemicarbazide condensed with salicylaldehyde derivations. Individual differences within the six investigated compounds arise from the property of the substituent. The geometry is characterized by a dihedral angle of between thiosemicarbazone core moiety and aromatic ring.

The energy of HOMO and LUMO of a molecule is an important quantum chemical descriptor, which plays a major role in chemical reactions [41]. According to the frontier molecular orbital theory of chemical reactivity, the formation of the transition state in chemical reactions is due to the interaction between the HOMO and LUMO orbitals of reacting species. In this work, the frontier orbitals HOMO and LUMO of the studied compounds were calculated at B3LYP/6-31G(d) level. The energy of the HOMO-LUMO orbitals of the studied compounds is shown in Table 2 and Figure S3. It can be found that **TSC-2** has an energy gap value of 4.26 eV, the lowest value, indicating that **TSC-2** has relatively high reactivity.

In order to evaluate the activity of an antioxidant via the hydrogen donating mechanism, bond dissociation enthalpy (BDE), which is related to the ability to donate a hydrogen atom, is assessed. The lowest BDE is defined for the relevant position where the easiest hydrogen atom abstraction for free radical scavenging reaction can take place [29,42]. The BDE values of molecules were calculated by using the B3LYP/6-31G(d) level of theory. There are three possible hydrogen bond breaking sites in our compounds, and the corresponding BDE values are listed in Table 2. The BDE values of N-H are in the range of 98.41–98.84 kcal/mol, significantly larger than the BDEs of N-H-N=C and O-H in molecules (80.86–86.54 kcal/mol). This indicates that the N-H is unfavorable to donating H atoms compared to the N-H-N=C and O-H groups. It is noteworthy that the calculated N-H-N=C and O-H BDEs of **TSC-2** are 80.86 and 81.24 kcal/mol, respectively, which are far smaller than other compounds. This means that **TSC-2** should have a stronger H-donating ability than other compounds; in other words, **TSC-2** has the highest scavenging radical activity. The calculated result is consistent with the experimental result. 

The optimized structures of the most stable radical for the evaluated compounds are shown in Figure 6. Table S2 reports the main geometrical parameters of radicals, including bond length and dihedral angle. It was found that when the H atom was abstracted translating to radical form, the radical was able to rearrange itself to assume the most stable conformation [29]. The obtained results show that a significant geometrical change in the generated radicals occurs in comparison to the neutral molecule. For example, in Figure 6, the dihedral angles of **TSC-2** changed from 59.41° to 0.65°—that is, from a non-planar structure to an almost planar structure. The dihedral angle of 0.65° allows a good electronic delocalization, which is favorable for the stability of the radical structure. 

**Figure 7:**
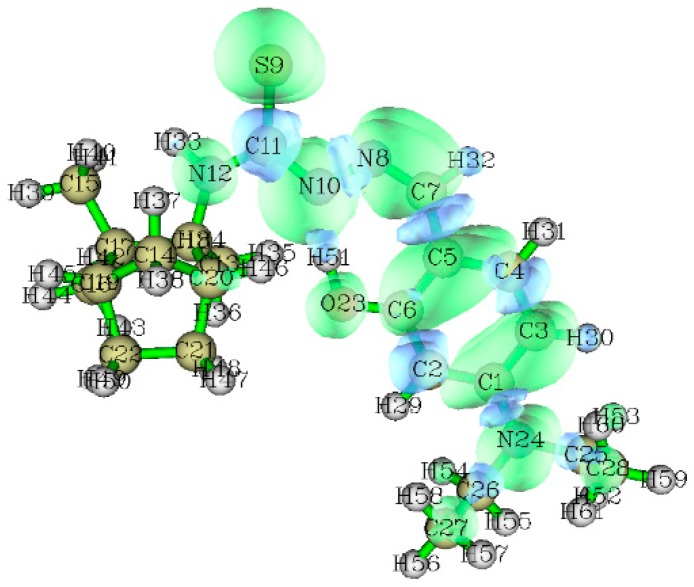
The Spin density distribution of **TSC-2** radical.

The difference in antioxidant activity was attributed to electron delocalization, which led to the stabilization of the radicals obtained after the H-atom abstraction. This result is made on the basis of an assumption that if electron delocalization exists in the parent molecule, it also exists in the corresponding radical. To better understand the relationship between electron delocalization and the reactivity of the radical, the electron delocalization should be examined. The spin density distribution offers a better insight into the delocalization of the unpaired electron and conjugation effects, which is a measure of the stability of the radicals [43]. The spin density distribution plot is presented in Figure 7.

As can be seen from Figure 7, **TSC-2** has more delocalized spin, which stabilizes the radical formed. These results suggest that the significantly enhanced radical scavenging activity of the compound **TSC-2** is attributed to the stabilization of the radical by both the electron-donating effect of the diethylamino group and radical delocalization over thiosemicarbazone core moiety and benzene rings resulting from the almost coplanar structure.

Next, the electron transfer mechanism was analyzed. The concepts of adiabatic ionization energy (AIE) have been applied successfully in the study of electron transfer reactions. AIE values describe the electron donation by a better ionization capacity and easier electron donation. The higher the AIE is, the more difficult an electron is to be removed. Molecules characterized by a low AIE are good electron donors [28,29]. In this work, the AIE of molecules were calculated using the B3LYP/6-311G(d) level of theory. The geometrical parameters of cation radicals are available in Table S3. The AIE is calculated and presented in Table 2. It should be noted that the AIE values have no notable difference, ranging from 5.41 to 5.56 eV. This means that the ability of electron removal from those compounds is not very different. In addition, the AIE value is greater than BDE, and hence the mechanism is not preferred. 

In this study, quantum chemical calculation was employed to illuminate **TSC-2** with the highest antioxidant activity on the basis of parameters containing frontier molecular orbitals, BDE, and the spin density distribution. The results suggest that the hydroxyl group, together with diethylamino group, play a synergistically antioxidative role for the studied compounds [44].

## 3. Materials and Methods

All the chemicals and reagents were obtained commercially and used without purification. 6-hydroxy-2,5,7,8-tetramethylchroman-2-carboxylic acid (Trolox) and 1,1-diphenyl-2-picrylhydrazyl (DPPH) were purchased from Sigma-Aldrich, Shanghai, China; 2,2′-azobis(2-amidinopropane) dihydrochloride (AAPH) and 2′,7′-dichlorofluorescin diacetate (DCFH-DA) were supplied by Macklin, Shanghai, China. Absorbance measurements were recorded on a SHIMASZU UV-2450 UV-Visible spectrophotometer (Shimadzu Corp., Tokyo, Japan). Fluorescence measurements were recorded on BioTek Cytation 5 multi-function Microplate Reader (BioTek Instruments, Inc., Winooski, VT, USA). ^1^H- and ^13^C-NMR spectra were recorded on a Bruker instrument (Bruker BioSpin, Rheinstetten, Germany) at 400 and 101MHz, respectively, using DMSO-d6 as solvent.

### 3.1. Synthesis of the Camphene-Based Thiosemicarbazones (**TSC-1~6**)

**Scheme 1:**
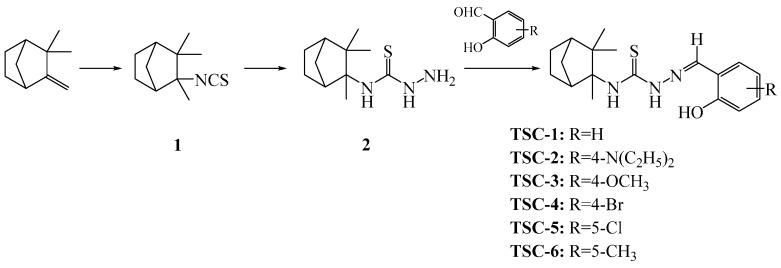
Synthesis and structures of camphene-based thiosemicarbazones.

The synthetic pathways and structural features of the compounds are outlined in Scheme 1.

Compounds 2-isocamphanyl isothiocyanate (**1**) and 4-(2′-isocamphanyl) thiosemicarbazide (**2**) were prepared according to the literature procedure [45]. The camphene-based thiosemicarbazones **TSC-1~6** were prepared according to the method described in reference [27]. The synthesized compounds were characterized by ^1^H-NMR and ^13^C-NMR in supporting information.

In ^1^H-NMR spectra, the thiosemicarbazone moiety contained the imine hydrogen (N=CH) and two NH. The hydrogen of N=CH is assigned as a singlet at 8.22–8.39 ppm, and the two NH chemical shifts appeared as two singlets at 11.02–11.45 ppm and 7.78–7.99 ppm. The hydrogen on the phenolic hydroxyl group was assigned as a singlet at 9.61–10.52 ppm, and aromatic hydrogens appeared at 6.10–7.61 ppm, depending on the substituent in the aromatic group. In the ^13^C-NMR spectra, the signals between 97.22 and 175.22 ppm were assigned resonance of aryl and unsaturated carbons. The most signals at 174.39–175.22 ppm were attributed to the carbon resonance of the thiocarbonyl group (C=S). The signal for C=N was observed at 136.10–140.07 ppm. All the signals are consistent with the structures. 

### 3.2. DPPH Radical Scavenging Assay

The DPPH radical scavenging capacity was performed at the fixed reaction time according to the published method with slight modification [46]. The assay was conducted using a microplate reader with a spectrophotometric detector.

To ensure complete dissolution, the test compounds were dissolved in a small volume of DMSO to generate a 10 mM stock solution and diluted to generate the final testing solution with ethanol. Different concentrations of DPPH solution were prepared to make a linear relationship between radical concentration and absorbance. The exact DPPH concentration was calculated from the calibration curve (Figure S4). The DPPH ethanol solution (68 µM) was prepared daily. The 225 µL ethanol solution of DPPH was added into the 25 µL of tested compounds solution with different concentrations (or 25 µL ethanol as blank control) on 96-well plates and allowed to react for one hour at room temperature in the dark. The absorbance was measured at 517 nm on a Microplate Reader. All tests and analyses were undertaken in three replicates and the results were averaged. The radical scavenging activity of the evaluated compounds was expressed as the percentage scavenging of free radical and was calculated using the following equation.

(1) $$\mathrm{Percentage}\;\mathrm{of}\;\mathrm{Scavenging}\;\left(\%\right)=\left[(A_{0}-A_{1}\right)/\left(A_{0}-A_{blank}\right)]\times 100,$$

Here, A_0_, A_1_, and A_blank_ represent the absorbance of DPPH in the absence or in the presence of antioxidant and blank. 

The effective concentration (EC_50_) value was defined as the efficient concentration required to decrease the preliminary DPPH radical concentration by 50%. The value of EC_50_ was expressed in terms of the molar ratio of antioxidant to DPPH and estimated by using a dose-response template from regression models in OriginPro 2019 soft.

### 3.3. Scavenging DPPH Kinetics Assay

The reaction of DPPH with antioxidants is basically a kinetic driving process. Thus, it is necessary to assess the kinetic behavior between the DPPH radical and the tested compounds. The experiments were carried out in excess of DPPH radical (the molar ratios of DPPH/antioxidant ranging from 1.5~15:1) in order to use up the H-donating capacity or the e-donating capacity of the tested compounds. The final concentration of DPPH was about 62 µM, while the tested compounds were added in the range 4–40 µM (final concentration).

The DPPH kinetic assay was adopted from the method described previously [47]. Briefly, 3900 µL of DPPH solution was added to the 100 µL of antioxidant solutions at different concentrations (or ethanol alone as a control). The recording of absorbance at 517nm was initiated immediately. Absorbance was recorded every 2.5 s for 120 min to generate reaction curves. The delay time between the addition of DPPH solution and the first absorbance reading was 6 s, and this was considered when plotting the absorbance–time graph. The decline in absorbance as a function of time is exponential in nature and plotted for different concentrations of antioxidants. The scavenging rate and percentage of remaining DPPH radical at infinite time were calculated according to the method described by Campos [48].

An empirical bi-exponential [49] Equation (2) was fitted to the date,

(2) y=A1e−xt1+A2e−xt2+y0

where the intercept y is constrained to equal A_0_, A_t_ is the absorbance at time t; A_1_ and A_2_ are the fast decay amplitude and slow decay amplitude, respectively; t_1_ and t_2_ are time constants of the fast and slow decays; y_0_ is remaining unreacted DPPH at infinite time. 

The bleaching rate is performed by the first derivative of Equation (3),

(3) −dAdt=A1/t1e−xt1+A2/t2e−xt2

Substituting x = 0 into the below Equation (4) offers an equation for the bleaching rate extrapolated to zero time,

(4) $$-{\left(\frac{\mathrm{dA}}{\mathrm{dt}}\right)}_{0}=A_{1}k_{1}+A_{2}k_{2},$$

A plot of ${\left(dA/dt\right)}_{0}$ against A_0_[AH] shows the bimolecular rate constant (k_b_) as the slope of the linear fit. The extrapolation to “infinite” time was chosen to avoid the uncertainty associated with the visual estimation of the steady-state conditions [50]. The total stoichiometry factor (*n*) was calculated according to the previously reported literature [49].

### 3.4. Peroxyl Radical Scavenging Capacity (PSC) Assay

The total antioxidant activity of the title compounds was determined using the PSC assay previously described [35] with a little modification. Trolox was used as a reference. The tested compounds and Trolox were diluted by 12% methylated β-cyclodextrin in ethanol to six different concentrations. In brief, 100 µL of samples or reference were added into a black 96-well microtiter plate, and then 100 µL of DCFH dye (33.06 µM) was added. The DCFH dye was prepared by hydrolyzing DCFH-DA with KOH solution (1 mM) for six min to remove the diacetate moiety before use; later, 50 µL of AAPH (40 mM) was added. The reaction proceeded for 36 min at 37 °C. Fluorescence intensity was monitored at the 485 nm excitation wavelength and 538 nm emission wavelength with a Microplate Reader. The control reactions used 12% methylated β-cyclodextrin ethanol solution. The areas under the average fluorescence–reaction time kinetic curves (AUC) for both the control and the tested compounds were integrated and used to calculate antioxidant activity in accordance with Equation (5):

(5) PSC unit=1−(SACA)

where SA and CA represent the AUC for a sample and the control reaction, respectively. The EC_50_ was also expressed as micromoles of Trolox equivalents per micromole of the evaluated compound.

### 3.5. Theoretical Calculations

According to the previously reported literature [28,30,51], generally, the main mechanisms of antioxidant action have been proposed and widely accepted, including hydrogen atom transfer (HAT), single electron transfer (SET) and metal chelation. The DPPH radical and ROO• radical could be scavenged by the tested compounds via the HAT mechanism or SET mechanism. 

In the HAT mechanism, the antioxidant becomes a radical after the free radical removes a hydrogen atom from the antioxidant. The BDE of N-H band or O-H band is an important parameter in evaluating the antioxidant action; the lower the BDE value, the easier the dissociation of the N-H or O–H bond. In the SET mechanism, the AIE is the most significant parameter for the scavenging activity evaluation; the lower the AIE value, the easier the electron abstraction. It is clear that as far as specific molecular properties are concerned, BDE and AIE are of particular importance to decide which mechanism is the favored one [42,52].

In addition, the HOMO-LUMO energy gap value is also an important parameter in determining the reactivity of a molecule. As the energy gap decreases, the more reactive the molecule will be and vice versa [41].

Base on the description above, in order to further understand the scavenging activity of the tested compounds, the theoretical calculation was performed at DFT level. The computational work was performed through the Gaussian 09 software package [39]. The geometry optimizations and frequency calculations of all the studied species were carried out using the M06-2X/Def2-SVP level and B3LYP/6-311G(d) level of theory, in conjunction with the polarizable continuum solvation (PCM) model [40] using ethanol as solvent. The frontier orbitals HOMO and LUMO of the studied compounds and the BDEs of molecules were calculated at the same level used for structural optimization according to the previously reported literature [30,42]. The AIEs of the tested compounds were computed using the B3LYP/6-311G(d) level of theory. Unrestricted calculations were used for the open-shell systems, such as radicals and radical cation species. To refine the energies, single-point calculations were performed on the optimized structures at the (U)B3LYP/6-31G(d) level.

All of the electron density analysis was conducted with the help of the Multiwfn software [53].

## 4. Conclusions

In conclusion, analysis of the antioxidant potential of six camphene-based thiosemicarbazones was performed by adopting experimental methods including the DPPH assay and the peroxyl radical scavenging capacity assay. The theoretical approach using DFT was employed to analyze the experimental results. According to the experimental results, all investigated compounds exhibited pronounced activity towards DPPH radical and peroxyl radical compared with Trolox. In particular, **TSC-2** exhibited the highest DPPH scavenging activity with the lowest EC_50_ (0.208 ± 0.004 mol/mole DPPH), as well as the highest bimolecular rate constant K_b_ (M^−1^ s^−1^), which is 1.18 times greater than Trolox. Meanwhile, in term of scavenging peroxyl radical assay, the experimental data indicated that **TSC-2** also possesses the highest peroxyl racial scavenging activity (1.27 µmol of Trolox equiv/µmol). Furthermore, further explanations for the experimental results have been determined by the DFT calculation. The analysis of the energy gap of HOMO-LUMO revealed that **TSC-2** has the lowest energy gap value and suggested that **TSC-2** possesses the best antioxidant activity of the studied compounds. The lowest BDE for **TSC-2** revealed that **TSC-2** likely acquired a high degree of antiradical scavenging by the HAT mechanism. The spin density distribution was used to analyze the stabilization of radical formed by HAT. The results suggest that the introduction of a strong electron-donating group (diethylamino group) in **TSC-2** leads to enhancing the radical scavenging activities by electronic delocalization. The analysis of the experimental and theoretical data presented in this paper supported the thesis that **TSC-2** is a promising molecule with high radical scavenging activity. In this work, the computed parameters gave some useful indication on free radical scavenging behavior of **TSC-2** by H-atom abstraction. In the future work, we need to determine the potential energy surfaces and the kinetic parameters which give qualitative and quantitative theoretical previsions to elucidate the antioxidant reaction mechanisms that the **TSC-2** compound follows.

## Figures and Tables

**Table 1:** Scavenging radical activities of compounds **TSC-1~6.**

Compounds	DPPH Assay				PSC Assay	
	EC_50_(µM) at Fixed Time ^1^	EC_50_ mol/mol DPPH Kinetic	Stoichiometric Factor (n) ^2^	Bimolecular Rate Constant ^3^ k_b_ (M^−1^ s^−1^)	EC_50_ (µM)	PSC Value (µmol of Trolox equiv/µmol)
**TSC-1**	0.411	0.308 ± 0.003	1.29 ± 0.23	16.16 ± 1.15	62.26 ± 1.65	0.34
**TSC-2**	0.208	0.208 ± 0.004	2.27 ± 0.13	4218.08 ± 551.26	16.78 ± 0.12	1.27
**TSC-3**	0.246	0.226 ± 0.002	2.27 ± 0.20	308.71 ± 19.91	37.78 ± 0.13	0.56
**TSC-4**	0.475	0.398 ± 0.006	1.21 ± 0.16	16.61 ± 0.75	34.62 ± 0.58	0.62
**TSC-5**	0.393	0.341 ± 0.015	1.33 ± 0.23	22.87 ± 2.32	43.33 ± 0.99	0.49
**TSC-6**	0.297	0.302 ± 0.015	1.44 ± 0.30	45.61 ± 4.13	48.38 ± 0.12	0.44
**Trolox**	0.241	0.249 ± 0.002	1.86 ± 0.12	3527.11 ± 103.95	21.38 ± 0.25	1

^1^ The fixed time was one hour; ^2^ Stoichiometric factor expresses the number of radical scavenged by one molecule of antioxidant; ^3^ The bimolecular rate constant (k_b_) was determined by the slope of a linear plot of ${\left(dA/dt\right)}_{0}$ against A_0_[AH].

**Table 2:** Rate constants(kb) for radical scavenging, HOMO-LUMO energy gap, bond dissociation enthalpy (BDE) and adiabatic ionization energy (AIE) values of compounds.

Compounds	BDE ^1^ (kcal/mol)			kb (M^−1^ s^−1^) ^2^	HOMO-LUMO Energy Gap (eV)	AIE (eV) ^3^
	NH	NH-N=CH	OH			
**TSC-1**	98.57	84.63	84.63	16.16	4.53	5.45
**TSC-2**	98.49	80.86	81.24	4218	4.26	5.41
**TSC-3**	98.59	85.33	85.92	308.71	4.67	5.49
**TSC-4**	98.84	86.48	83.53	16.61	4.48	5.56
**TSC-5**	98.51	86.54	85.47	22.87	4.53	5.56
**TSC-6**	98.41	85.99	83.51	45.61	4.54	5.46

^1^BDE is bond dissociation enthalpy; ^2^k_b_ represented the bimolecular rate constant, which was determined by the slope of a linear plot of ${\left(dA/dt\right)}_{0}$ against A_0_[AH]; represented; ^3^AIE is adiabatic ionization energy.

## Supplementary Materials

The following are available online, Figure S1: (A)Reaction kinetics of DPPH radical with TSC-1, TSC-4, TSC-5, TSC-6, and Trolox; (B) %DPPH radical remaining at infinite time at the different concentration of TSC-1, TSC-4, TSC-5, TSC-6 and Trolox, Figure S2: Time kinetics plots of the TSC-1, TSC-4, TSC-5, TSC-6 inhibition of DCFH oxidation by AAPH. (the inset is dose-response plot); Figure S3: Optimized ground state structures and the orbital distribution and energy (eV) of HOMO and LUMO for the studied camphene-based thiosemicarbazone; Figure S4: Calibration curve of DPPH in ethanol at 517 nm; Figure S5~S10: 1H-NMR and 13C-NMR of TSC-1~6; Table S1 Selected bond lengths and dihedral angles of the neutral forms of thiosemicarbazone in ethanol solution Table S2. Selected bond lengths and dihedral angles of the radicals of thiosemicarbazone in ethanol solution; Table S3. Selected bond lengths and dihedral angles of the cations radical of thiosemicarbazone.
