# Supplementary material for: Antioxidant Properties of Camphene-Based Thiosemicarbazones: Experimental and Theoretical Evaluation
Source: Molecules. 2020 Mar 6;25(5):1192. doi: 10.3390/molecules25051192 (PMC7179440; doi:10.3390/molecules25051192)
Supplement: Supplementary file 1 [file molecules-25-01192-s001.pdf]

Supporting Information

Antioxidant Properties of Camphene-Based Thiosemicarbazones: Experimental and Theoretical Evaluation

Lijuan Yang [1], Haochuang Liu [1], Dasha Xia [2], and Shifa Wang [1,3,] *

[1] College of Chemical Engineering, Nanjing Forestry University, Nanjing, 210037, China; lijuan_yang@163.com; 1076943403@qq.com
[2] Hangzhou Yanqu Information Technology Co., Ltd., Hangzhou, 310012, China; xiadasha@hotmail.com
[3] Co-Innovation Center of Efficient Processing and Utilization of Forest Resources, Nanjing Forestry University, Nanjing, 210037, China;
* Correspondence: wangshifa65@163.com; Tel.: +86-25-8542-8369

Contents

- **Part 1 Result and discussion section**

Figure S1. Reaction kinetics of DPPH radical with **TSC-1(A)**, **TSC-4(C), TSC-5(E)**, **TSC-6(G)** and **Trolox**(I); %DPPH radical remaining at infinite time at the different concentration of **TSC-1(B)**, **TSC-4(D)**, **TSC-5(F)**, **TSC-6(H)** and **Trolox(J)**;

Figure S2. Time kinetics plots of **TSC-1(A)**, **TSC-4(B)**, **TSC-5(C)**, **TSC-6(D)** and **Trolox (E)** inhibition of DCFH oxidation by AAPH. (the inset is dose-response plot)

Figure S3. Optimized ground state structures and the orbital distribution and energy (eV) of HOMO and LUMO for the studied camphene-based thiosemicarbazone.

Figure S4. Calibration curve of DPPH in ethanol at 517nm.

Table S1 Selected bond lengths and dihedral angles of the neutral forms of thiosemicarbazone in ethanol solution

Table S2. Selected bond lengths and dihedral angles of the radicals of thiosemicarbazone in ethanol solution

Table S3. Selected bond lengths and dihedral angles of the cations radical of thiosemicarbazone

- **Part 2 Synthesis section**

Figure S5~10. ^{1}H NMR (**A**) and ^{13}C NMR (**B**) spectrum of **TSC-1~6**

- **Part 3 Cartesian coordinates of the investigated compounds**

Part 1 Result and discussion section

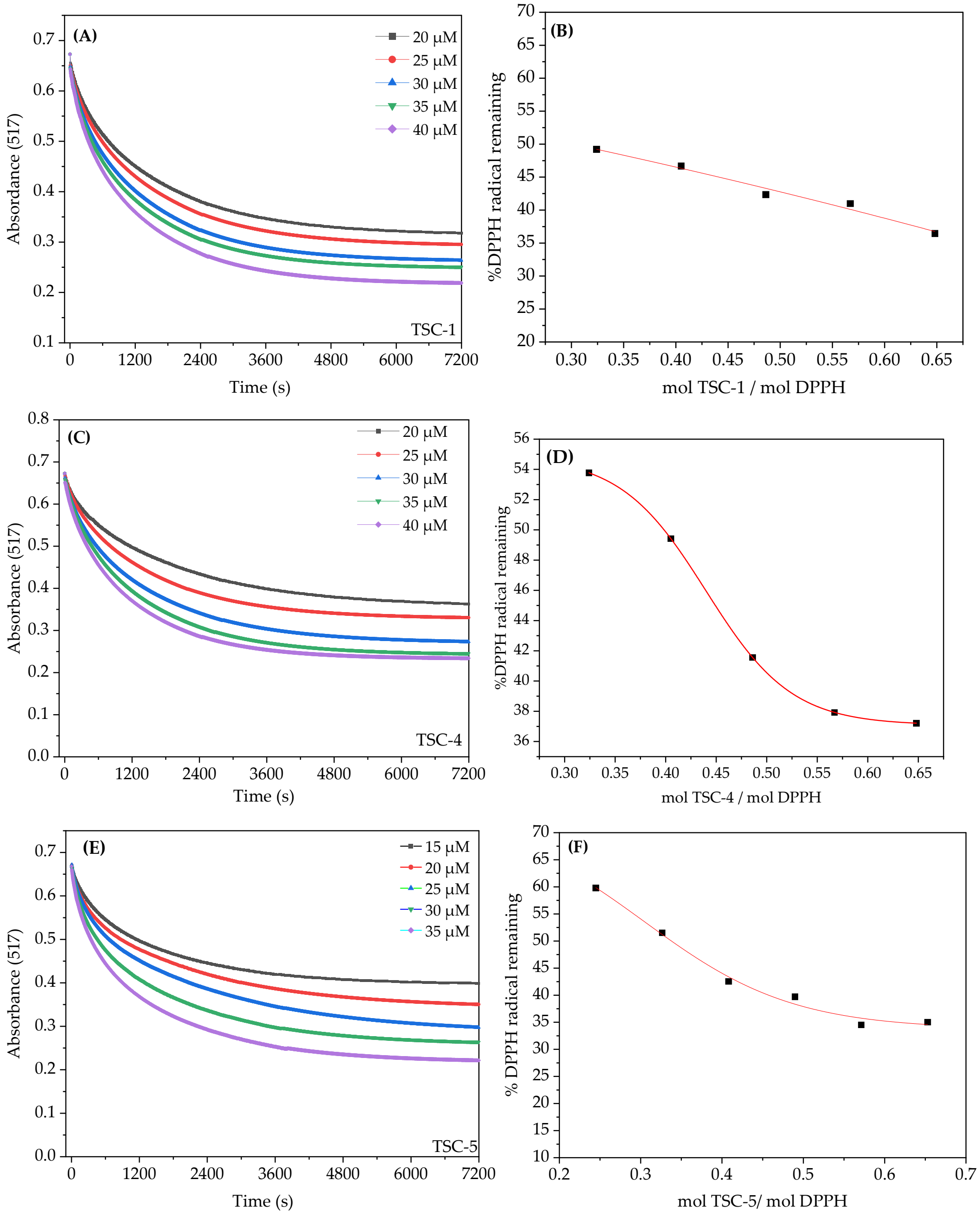

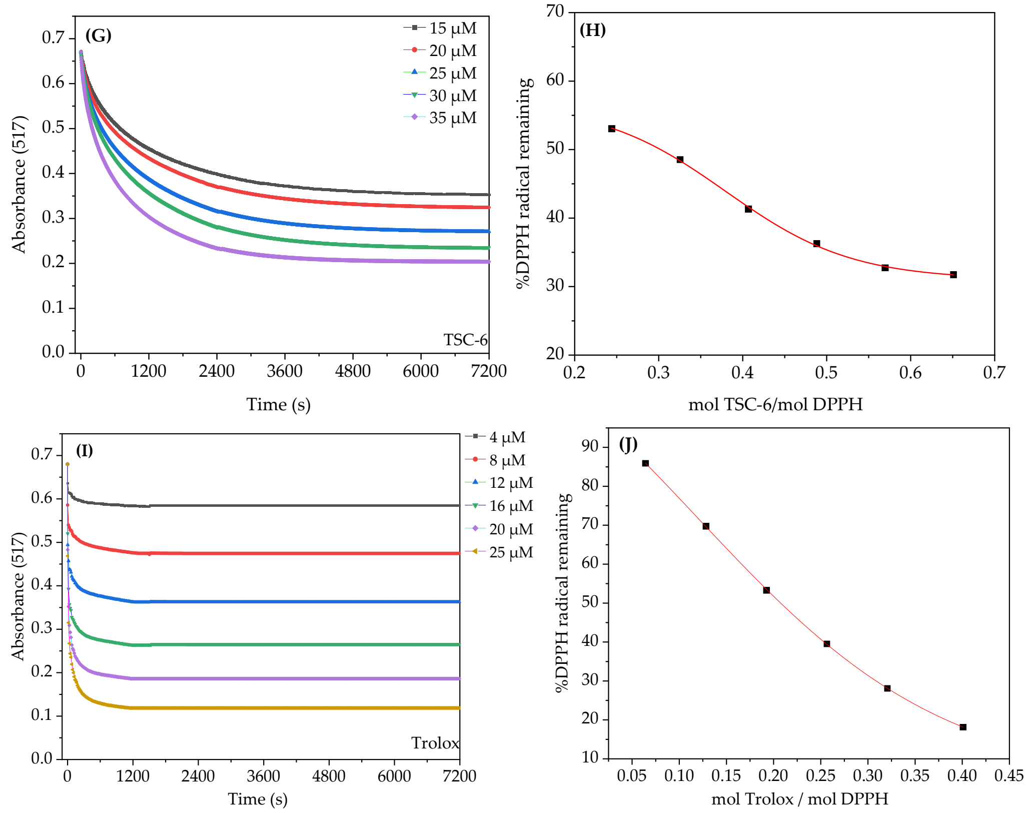

Figure S1. Reaction kinetics of DPPH radical with **TSC-1(A)**, **TSC-4(C)**, **TSC-5(E)**, **TSC-6(G)** and **Trolox**(I); %DPPH radical remaining at infinite time at the different concentration of **TSC-1(B)**, **TSC-4(D)**, **TSC-5(F)**, **TSC-6(H)** and **Trolox(J)**;

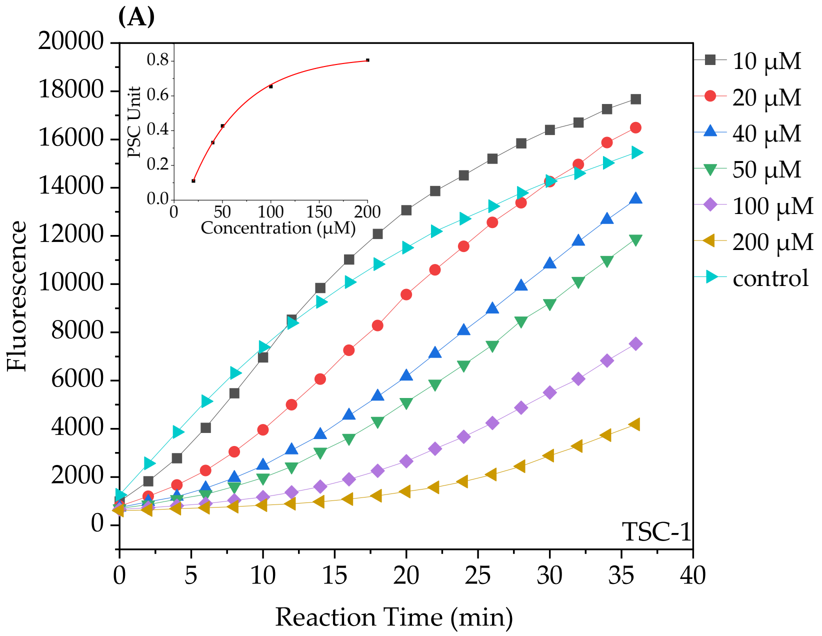

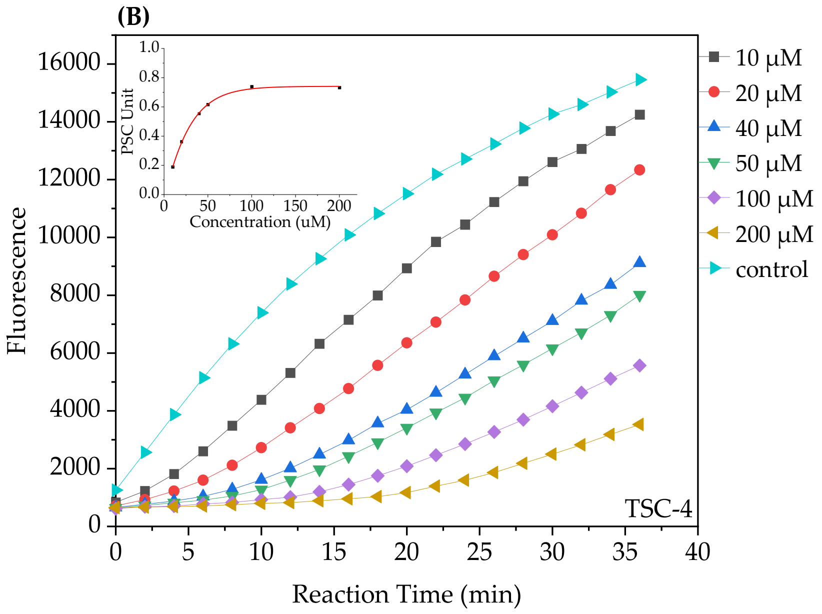
(B)
Fluorescence
Reaction Time (min)
PSC Unit
Concentration (uM)
10 μM
20 μM
40 μM
50 μM
100 μM
200 μM
control
TSC-4

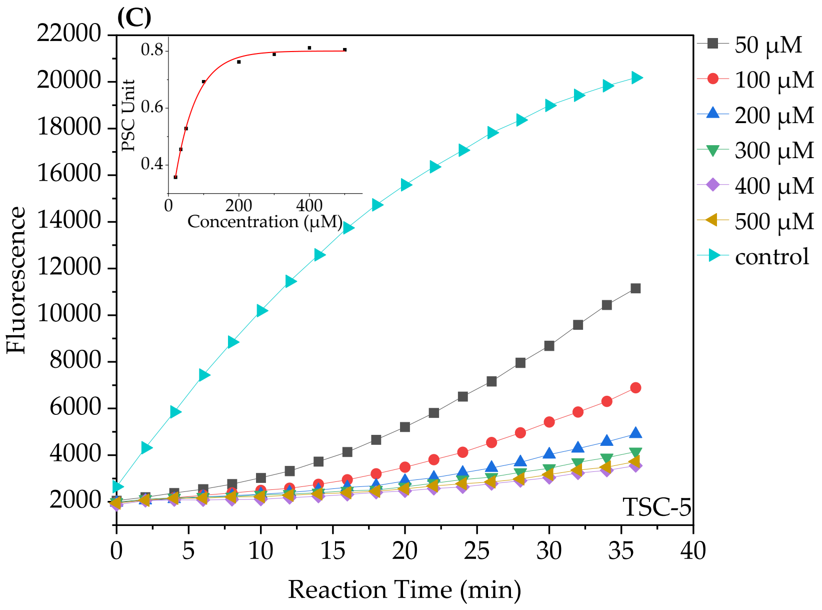
(C)
Fluorescence
Reaction Time (min)
PSC Unit
Concentration (μM)
50 μM
100 μM
200 μM
300 μM
400 μM
500 μM
control
TSC-5

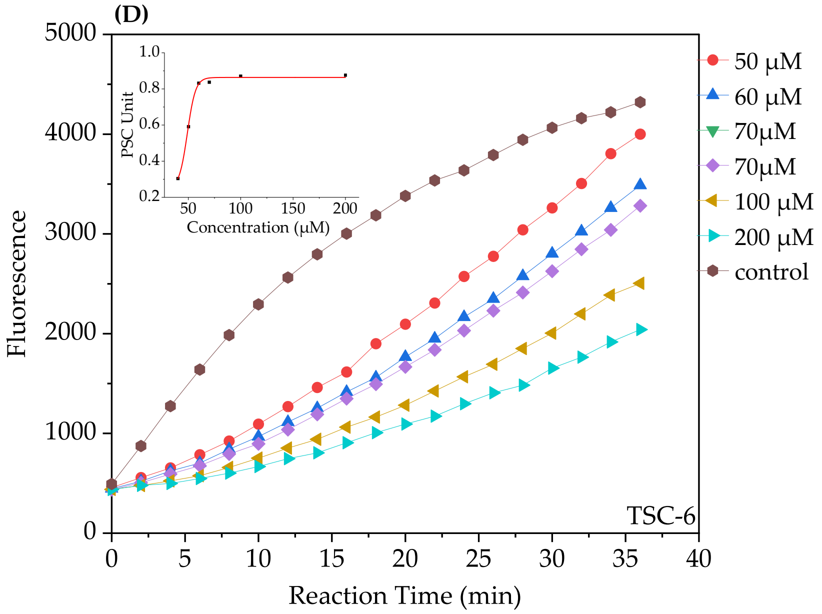

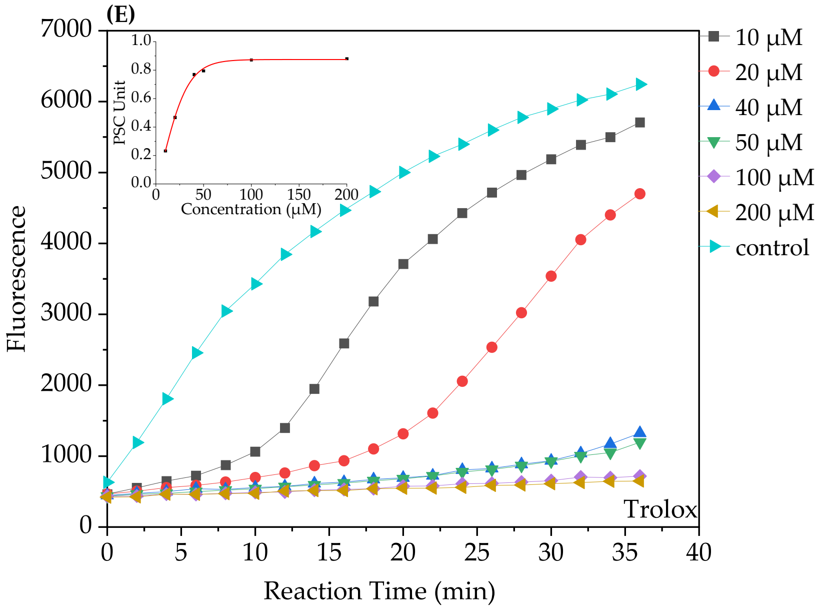

Figure S2. Time kinetics plots of **TSC-1(A)**, **TSC-4(B)**, **TSC-5(C)**, **TSC-6(D)** and **Trolox (E)** inhibition of DCFH oxidation by AAPH. (the inset is dose-response plot)

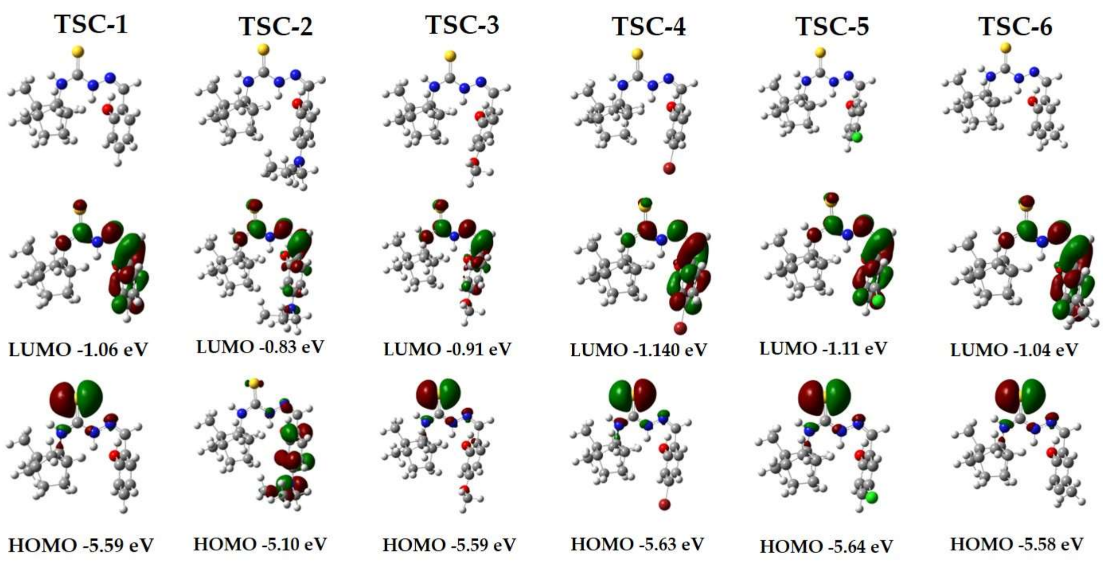

Figure S3 . Optimized ground state structures and the orbital distribution and energy (eV) of HOMO and LUMO for the studied camphene-based thiosemicarbazone

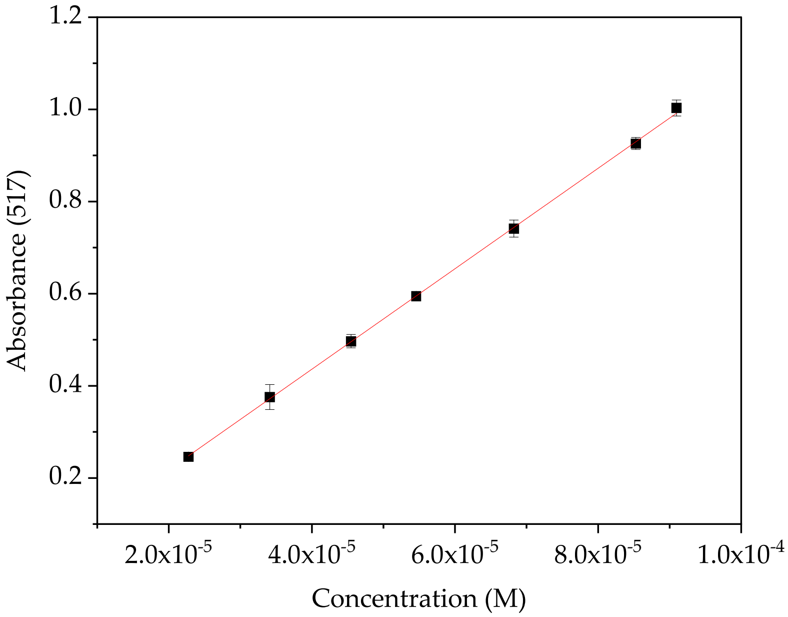

Figure S4. Calibration curve of DPPH in ethanol at 517nm. The exact initial DPPH concentration was calculated from the Eq. (A_{517} =10902× [DPPH], R^2 = 0.99998, n = 7). The molar absorbance coefficient (ε=10902M^{-1}) of DPPH in ethanol was also calculated based on the calibration curve because DPPH is sufficiently stable in ethanol [1].

Table S1. Selected bond lengths and dihedral angles of the neutral forms of thiosemicarbazone in ethanol solution.

Bond length (Å)	TSC-1	TSC-2	TSC-3	TSC-4	TSC-5	TSC-6
H-O(23)	0.970	0.970	0.970	0.971	0.971	0.970
H-N(12)	1.013	1.013	1.013	1.014	1.013	1.013
H-N(10)	1.019	1.018	1.017	1.018	1.017	1.019
O(23)-C(6)	1.353	1.356	1.352	1.895	1.351	1.355
N(12)-C(11)	1.345	1.347	1.347	1.348	1.346	1.345
C(11)-S(9)	1.683	1.684	1.683	1.346	1.682	1.684
C(11)-N(10)	1.370	1.366	1.366	1.682	1.369	1.37
N(10)-N(8)	1.348	1.351	1.349	1.369	1.346	1.348
N(8)-C(7)	1.282	1.284	1.283	1.347	1.282	1.282
C(7)-C(5)	1.486	1.482	1.488	1.282	1.49	1.486
C(6)-C(5)	1.408	1.408	1.411	1.488	1.407	1.404
C(5)-C(4)	1.403	1.403	1.397	1.407	1.4	1.404
Dihedral angle (°)	TSC-1	TSC-2	TSC-3	TSC-4	TSC-5	TSC-6
N(12)-C(11)-N(10)-N(8)	-170.65	-168.67	-172.16	-170.62	-170.07	-169.79
C(11)-N(10)-N(8)-C(7)	174.07	171.93	169.33	171.57	171.42	172.70
N(8)-C(7)-C(5)-C(6)	59.81	59.41	64.92	62.52	65.94	59.59

Table S2. Selected bond lengths and dihedral angles of the radicals of thiosemicarbazone in ethanol solution

Bond length (Å)	TSC-1	TSC-2	TSC-3	Bond length (Å)	TSC-4	TSC-5	TSC-6
H-O(23)	1.023	1.035	1.026	H-N(12)	1.014	1.014	1.013
H-N(12)	1.014	1.013	1.014	H-N(10)	1.027	1.038	1.037
O(23)-C(6)	1.333	1.319	1.319	O(23)-C(6)	1.328	1.248	1.25
N(12)-C(11)	1.331	1.344	1.34	N(12)-C(11)	1.33	1.342	1.343
C(11)-S(9)	1.765	1.687	1.681	C(11)-S(9)	1.765	1.682	1.684
C(11)-N(10)	1.310	1.397	1.404	C(11)-N(10)	1.311	1.384	1.379
N(10)-N(8)	1.360	1.3	1.297	N(10)-N(8)	1.359	1.319	1.325
N(8)-C(7)	1.278	1.329	1.327	N(8)-C(7)	1.278	1.296	1.293
C(7)-C(5)	1.478	1.422	1.433	C(7)-C(5)	1.477	1.457	1.463
C(6)-C(5)	1.424	1.445	1.439	C(6)-C(5)	1.425	1.469	1.467
C(5)-C(4)	1.412	1.432	1.425	C(5)-C(4)	1.41	1.404	1.402
Dihedral angle (°)	TSC-1	TSC-2	TSC-3	Dihedral angle (°)	TSC-4	TSC-5	TSC-6
N(12)-C(11)-N(10)-N(8)	-179.37	-171.11	-158.90	N(12)-C(11)-N(10)-N(8)	-179.56	-179.13	-179.45
C(11)-N(10)-N(8)-C(7)	-177.67	-179.32	177.61	C(11)-N(10)-N(8)-C(7)	179.24	179.902	179.40
N(8)-C(7)-C(5)-C(6)	-6.28	0.65	5.915	N(8)-C(7)-C(5)-C(6)	1.52	0.367	1.49

Table S3. Selected bond lengths and dihedral angles of the cation radicals of thiosemicarbazone

Bond length (Å)	TSC-1	TSC-2	TSC-3	TSC-4	TSC-5	TSC-6
H-O(23)	0.970	0.968	0.967	0.967	0.967	0.969
H-N(12)	1.012	1.008	1.011	1.012	1.012	1.012
H-N(10)	1.026	1.013	1.015	1.015	1.016	1.027
O(23)-C(6)	1.386	1.356	1.367	1.362	1.364	1.388
N(12)-C(11)	1.314	1.342	1.314	1.312	1.312	1.315
C(11)-S(9)	1.756	1.701	1.755	1.754	1.755	1.756
C(11)-N(10)	1.343	1.382	1.343	1.345	1.345	1.342
N(10)-N(8)	1.369	1.347	1.372	1.369	1.369	1.369
N(8)-C(7)	1.281	1.296	1.284	1.28	1.28	1.281
C(7)-C(5)	1.47	1.459	1.462	1.471	1.475	1.469
C(6)-C(5)	1.407	1.428	1.415	1.409	1.408	1.404
C(5)-C(4)	1.408	1.417	1.407	1.406	1.406	1.409
Dihedral angle (°)	TSC-1	TSC-2	TSC-3	TSC-4	TSC-5	TSC-6
N(12)-C(11)-N(10)-N(8)	179.191	-163.411	-175.761	-175.978	-176.1	179.105
C(11)-N(10)-N(8)-C(7)	173.712	-175.841	-176.969	-179.001	178.71	172.573
N(8)-C(7)-C(5)-C(6)	34.606	43.234	42.906	47.853	49.89	32.866

Part 2 Synthesis section of the camphene-based thiosemicarbazones

The mixture of compound **2** (1 mmol) and substituted salicylaldehyde (1 mmol) was refluxed in 15 mL of absolute ethanol in the presence of the hydrochloric acid. The reaction process was monitored with TLC, and the crude products were purified by recrystallization in ethanol.

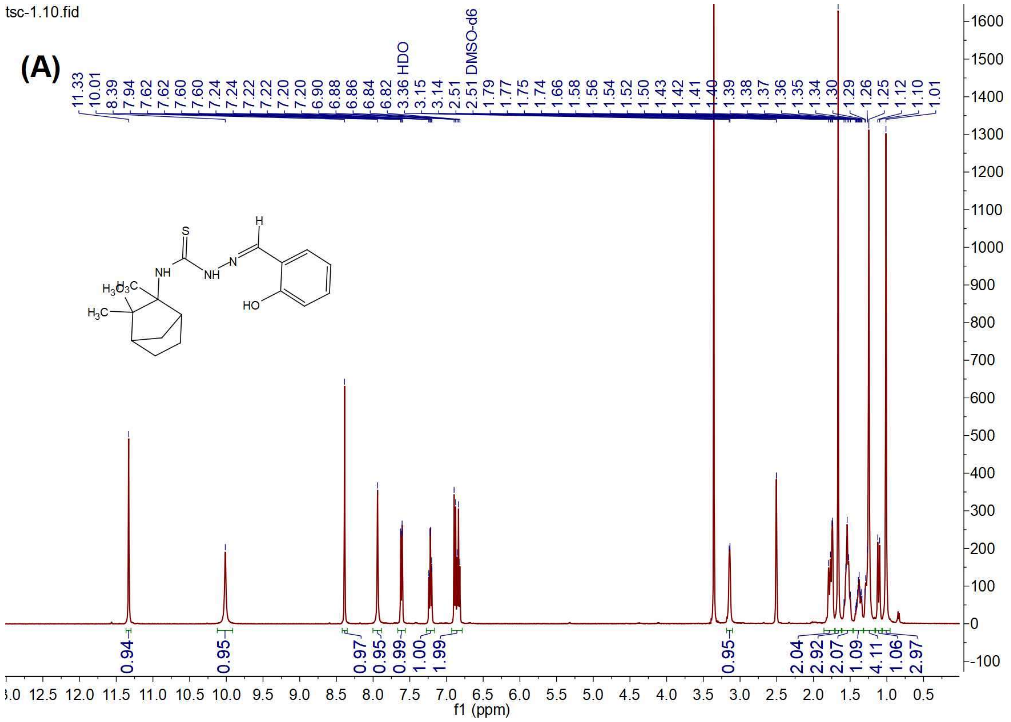

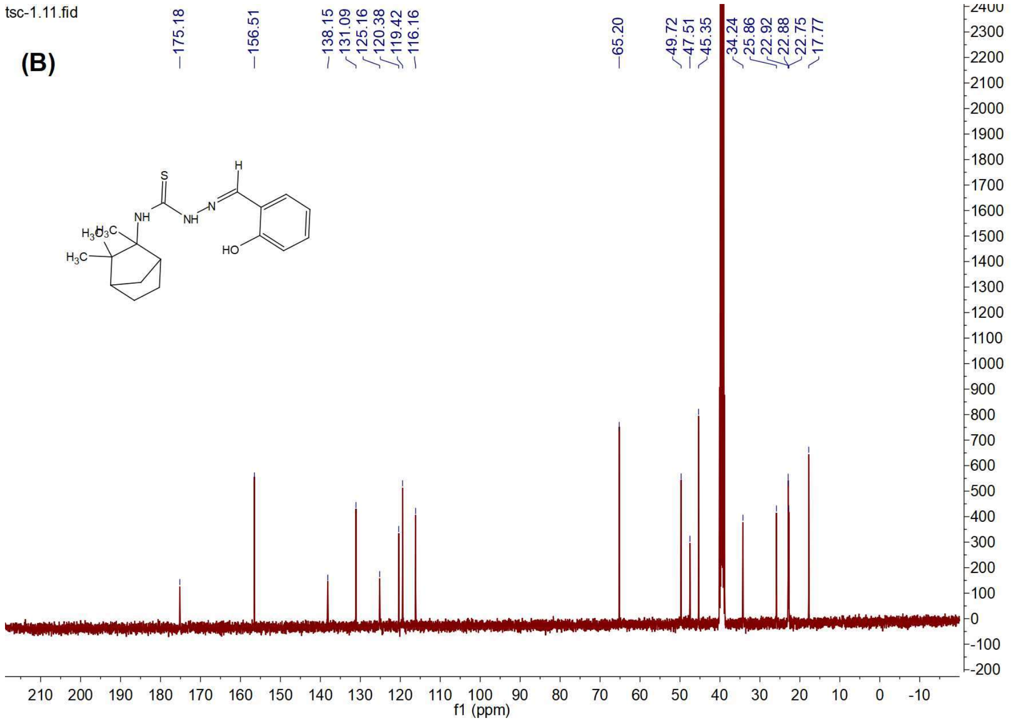

Figure S5. ^{1}H NMR (**A**) and ^{13}C NMR (**B**) spectrum of **TSC-1**

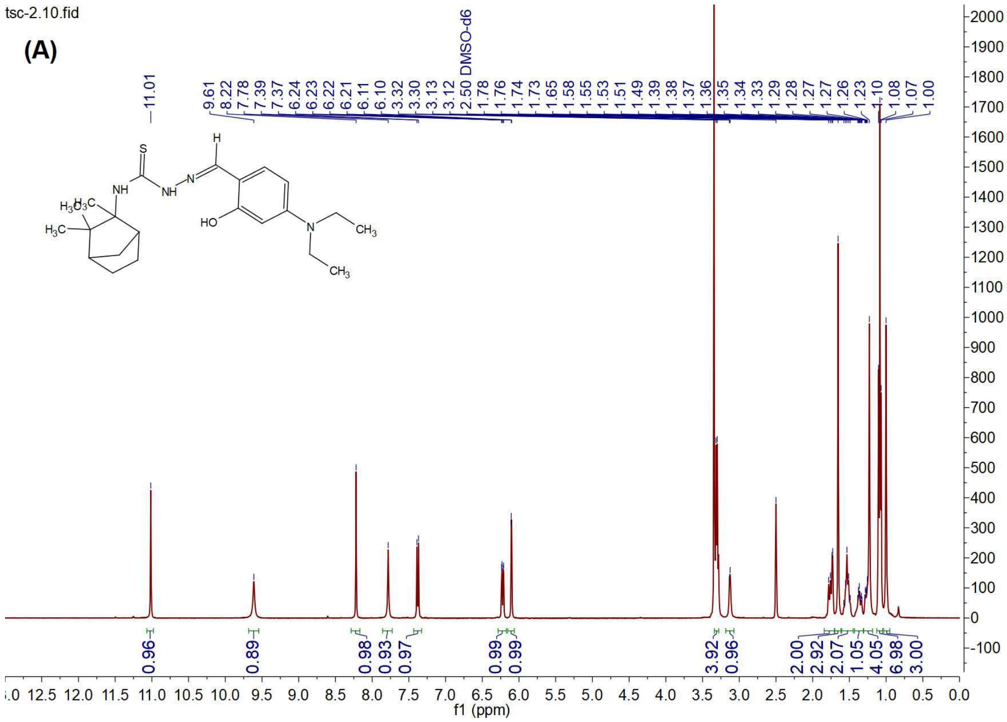

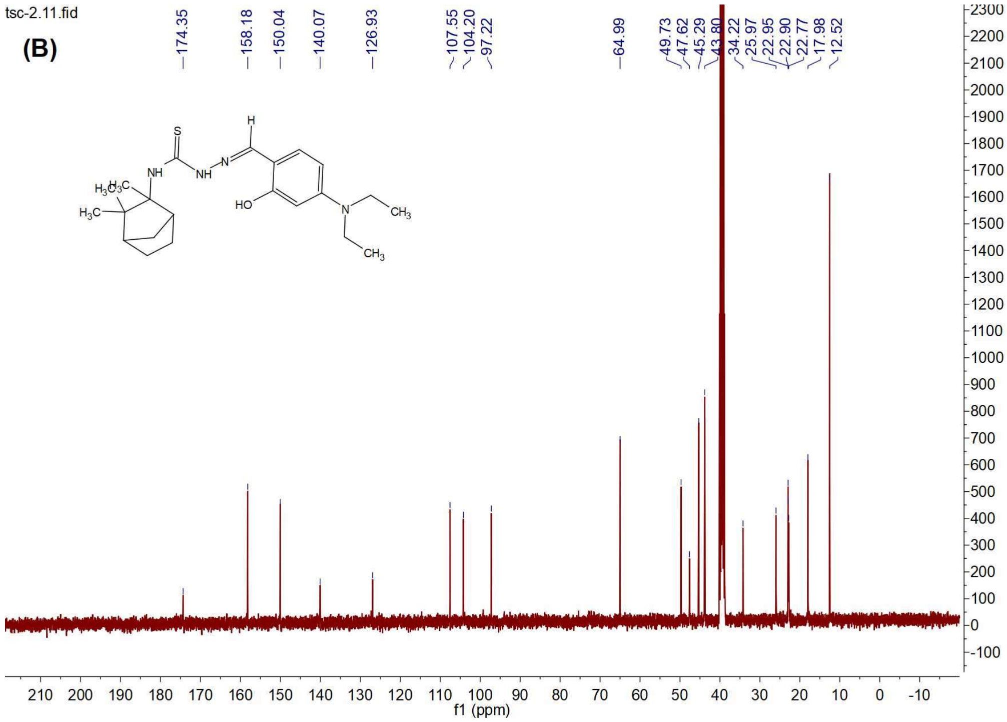

Figure S6. ^{1}H NMR (**A**) and ^{13}C NMR (**B**) of **TSC-2**

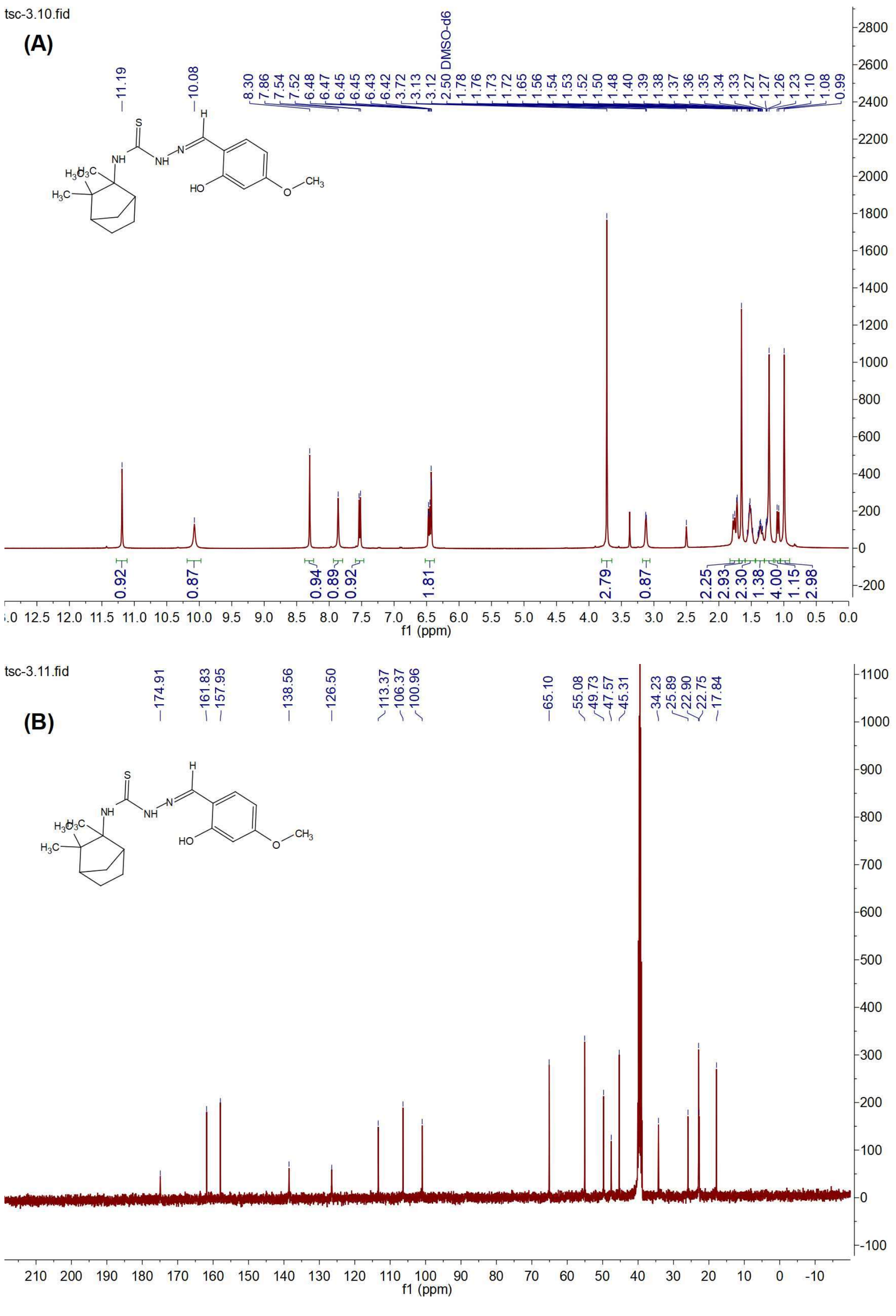

Figure S7. ¹H NMR (**A**) and ¹³C NMR (**B**) of **TSC-3**

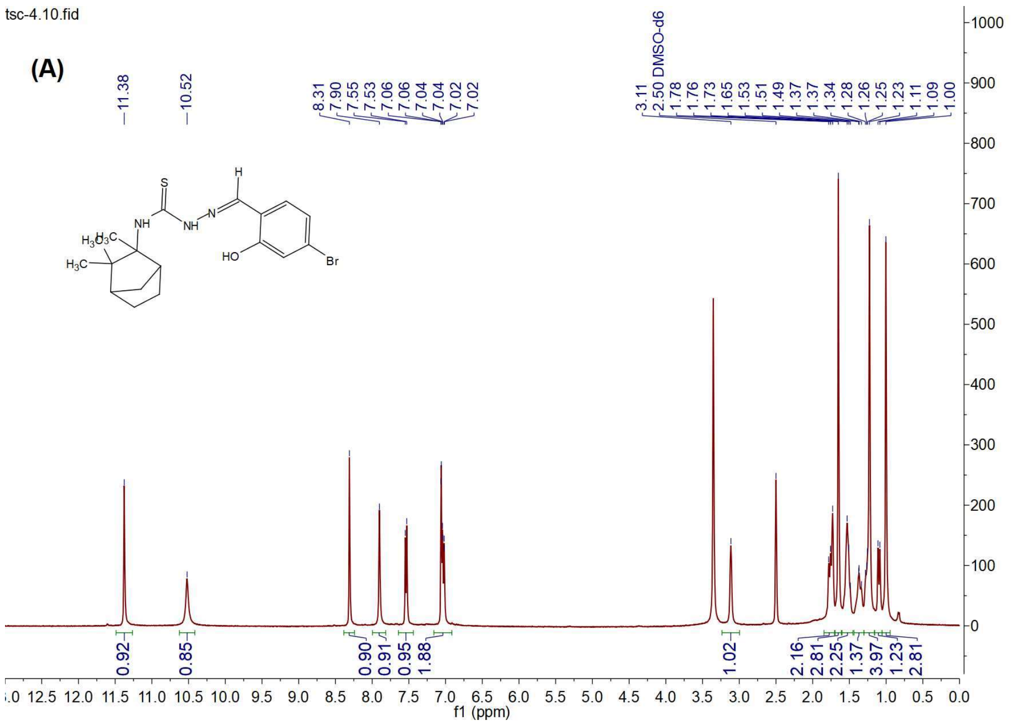

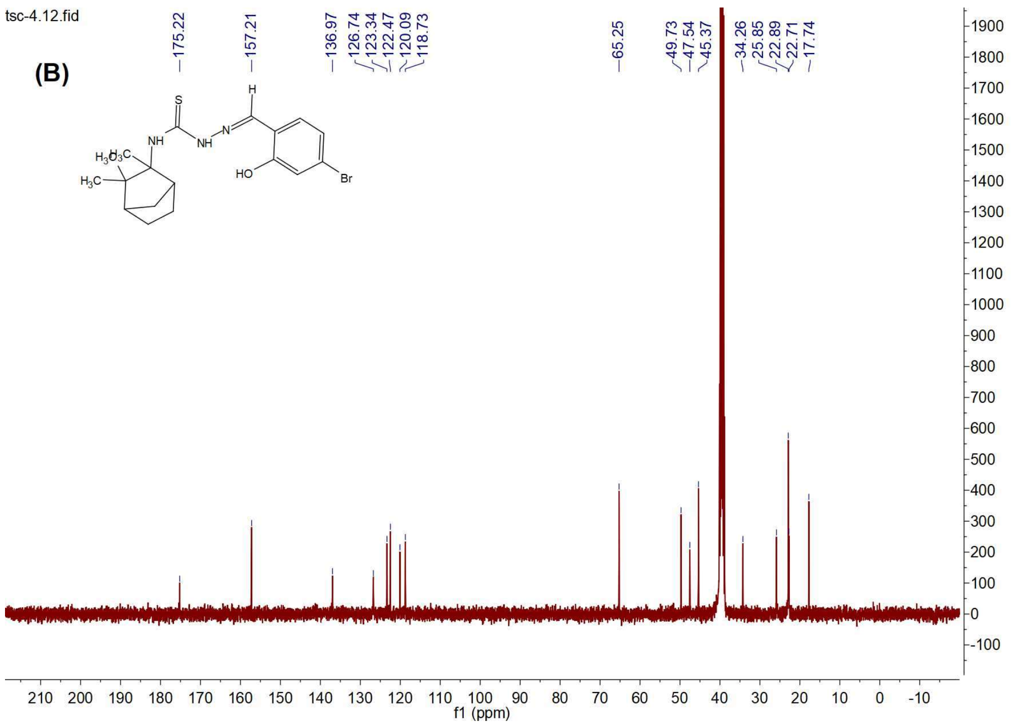

Figure S8. ^{1}H NMR (**A**) and ^{13}C NMR (**B**) of **TSC-4**

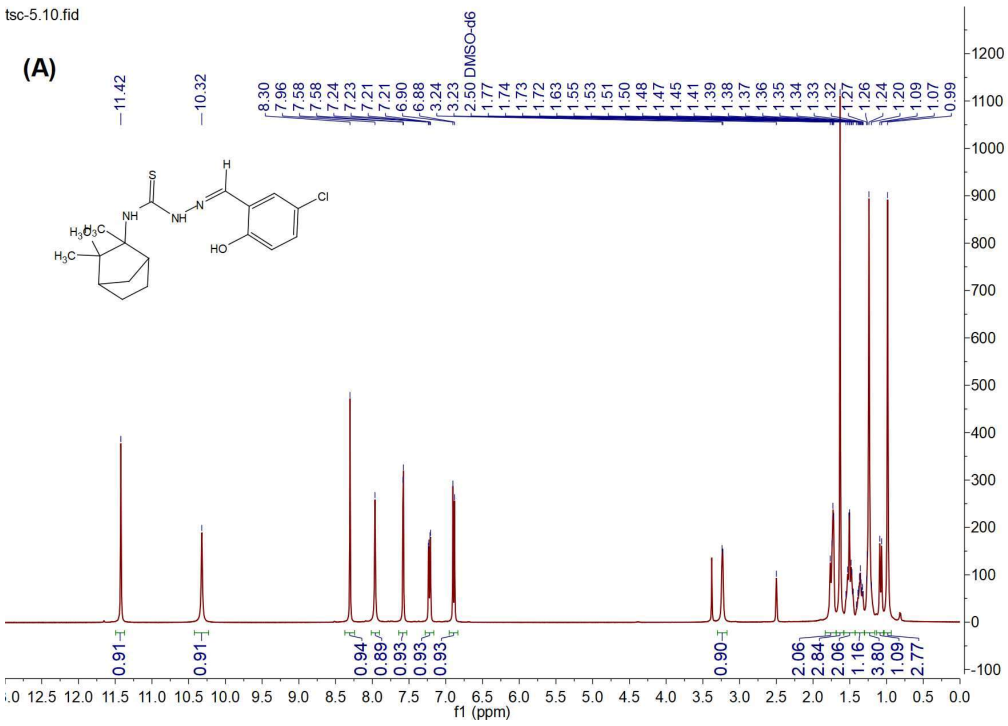

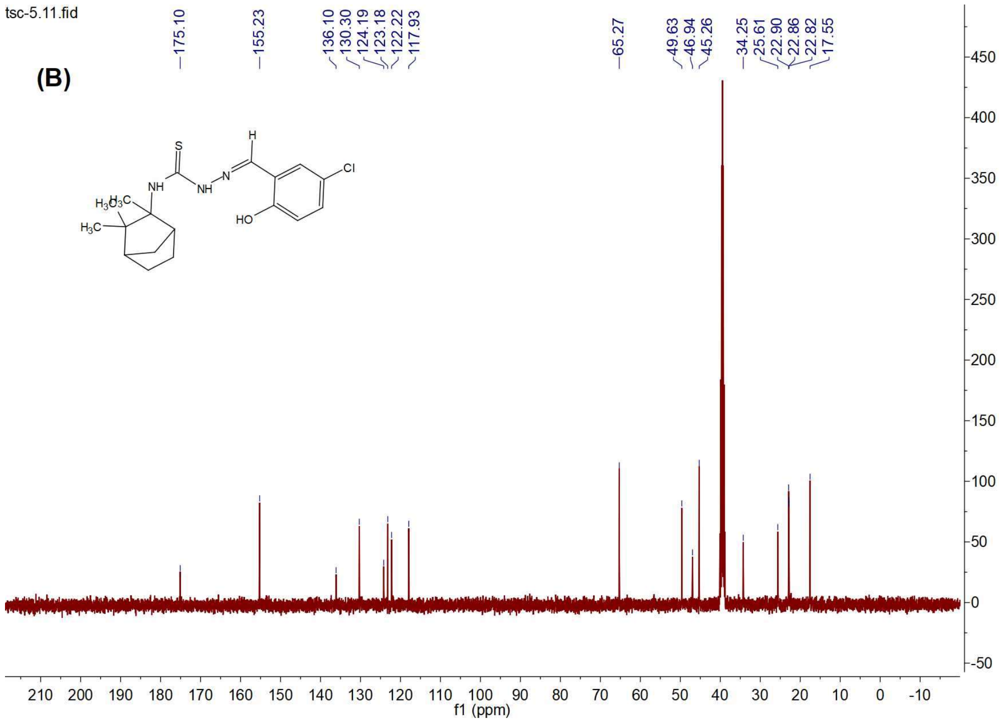

Figure S9. ^{1}H NMR (**A**) and ^{13}C NMR (**B**) of **TSC-5**

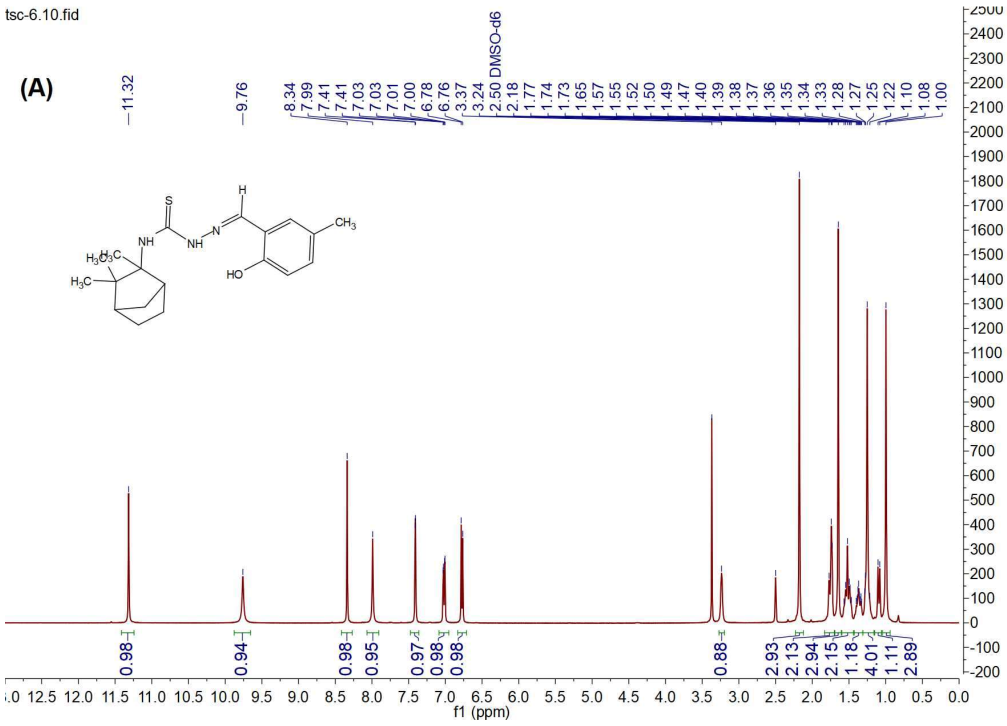

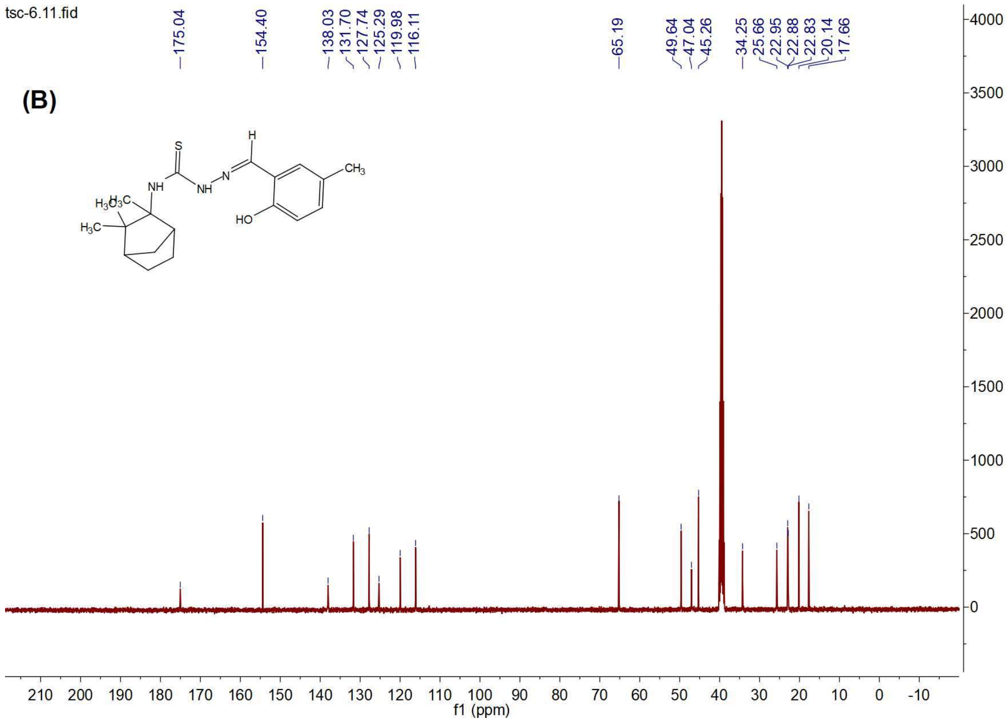

Figure S10. ^{1}H NMR (**A**) and ^{13}C NMR (**B**) of **TSC-6**

2-Hydroxybenzaldehyde-4-(2′-isocamphanyl) thiosemicarbazone White solid; yield 93.3%; ^{1}H NMR (400 MHz, DMSO-d6) δ 11.33 (s, 1H, NH), 10.01 (s, 1H, OH), 8.39 (s, 1H, N=CH), 7.94 (s, 1H, NH), 7.61 (dd, J = 7.8, 1.6 Hz, 1H, Ar-H), 7.22 (td, J = 7.8, 1.7 Hz, 1H, Ar-H), 6.93 – 6.79 (m, 2H, Ar-H), 3.14 (d, J = 4.3 Hz, 1H), 1.76 (dd, J = 15.2, 7.0 Hz, 2H), 1.66 (s, 3H), 1.53 (q, J = 9.2, 8.4 Hz, 2H), 1.38 (dd, J = 15.5, 11.8, 4.3 Hz, 1H), 1.25 (s, 4H), 1.11 (d, J = 10.1 Hz, 1H), 1.01 (s, 3H).

^{13}C NMR (101 MHz, DMSO-d6) δ 175.18(C=S), 156.51, 138.15(C=N), 131.09, 125.16, 120.38, 119.42, 116.16, 65.20, 49.72, 47.51, 45.35, 34.24, 25.86, 22.92, 22.88, 22.75, 17.77 (Figure S5).

2-hydroxy-4-(N,N-diethylamino) benzaldehyde-4-(2′-isocamphanyl) thiosemicarbazone (**TSC-2**). Yellow solid; yield 81.35%; ^{1}H NMR (400 MHz, DMSO-d6) δ 11.01 (s, 1H, NH), 9.61 (s, 1H, OH), 8.22 (s, 1H, N=CH), 7.78 (s, 1H, NH), 7.38 (d, J = 8.9 Hz, 1H, Ar-H), 6.22 (dd, J = 8.9, 2.4 Hz, 1H, Ar-H), 6.10 (d, J = 2.5 Hz, 1H, Ar-H), 3.31 (d, J = 7.1 Hz, 4H, 2CH2), 3.13 (d, J = 4.2 Hz, 1H), 1.84 – 1.70 (m, 2H), 1.65 (s, 3H), 1.52 (q, J = 9.1, 8.5 Hz, 2H), 1.36 (ddt, J = 11.4, 7.3, 4.3 Hz, 1H), 1.23 (s, 4H), 1.09 (t, J = 7.0 Hz, 7H), 1.00 (s, 3H). ^{13}C NMR (101 MHz, DMSO-d6) δ 174.35(C=S), 158.18, 150.04, 140.07(C=N), 126.93, 107.55, 104.20, 97.22, 64.99, 49.73, 47.62, 45.29, 43.80, 34.22, 25.97, 22.95, 22.90, 22.77, 17.98, 12.52 (Figure S6).

2-hydroxy-4-methoxybenzaldehyde-4-(2′-isocamphanyl) thiosemicarbazone (**TSC-3**). White solid; yield 70.06%; ^{1}H NMR (400 MHz, DMSO-d6) δ 11.19 (s, 1H, NH), 10.08 (s, 1H, OH), 8.30 (s, 1H, N=CH), 7.86 (s, 1H, NH), 7.53 (d, J = 8.7 Hz, 1H, Ar-H), 6.52 – 6.38 (m, 2H, Ar-H), 3.72 (s, 3H, OCH3), 3.12 (d, J = 4.3 Hz, 1H), 1.74 (dd, J = 18.8, 7.0 Hz, 2H), 1.65 (s, 3H), 1.59 – 1.44 (m, 2H), 1.36 (tq, J = 11.8, 4.6, 3.7 Hz, 1H), 1.23 (s, 4H), 1.09 (d, J = 10.0 Hz, 1H), 0.99 (s, 3H). ^{13}C NMR (101 MHz, DMSO-d6) δ 174.91(C=S), 161.83, 157.95, 138.56(C=N), 126.50, 113.37, 106.37, 100.96, 65.10, 55.08, 49.73, 47.57, 45.31, 34.23, 25.89, 22.90, 22.75, 17.84 (Figure S7).

2-hydroxy-4-bromobenzaldehyde-(2′-isocamphanyl) thiosemicarbazone ***(TSC-4)***. Yellow solid; yield 76.43%; ^{1}H NMR (400 MHz, DMSO-d_6) δ 11.38 (s, 1H, NH), 10.52 (s, 1H, OH), 8.31 (s, 1H, N=CH), 7.90 (s, 1H, NH), 7.54 (d, *J* = 8.4 Hz, 1H, Ar-H), 7.09 – 6.99 (m, 2H, Ar-H), 3.11 (s, 1H), 1.81 – 1.70 (m, 2H), 1.65 (s, 3H), 1.53 (s, 2H), 1.50 (d, *J* = 8.8 Hz, 0H), 1.37 (s, 1H), 1.35 (d, *J* = 13.5 Hz, 0H), 1.31 – 1.23 (m, 1H), 1.23 (s, 3H), 1.10 (d, *J* = 10.0 Hz, 1H), 1.00 (s, 3H). ^{13}C NMR (101 MHz, DMSO-d_6) δ 175.22(C=S), 157.21, 136.97(C=N), 126.74, 123.34, 122.47, 120.09, 118.73, 65.25, 49.73, 47.54, 45.37, 34.26, 25.85, 22.89, 22.71, 17.74 (Figure S8)

2-hydroxy-5-chlorobenzaldehyde-4-(2′-isocamphanyl) thiosemicarbazone **(TSC-5)**. Faint yellow soli; yield 74.37%; ^{1}H NMR (400 MHz, DMSO-d_6) δ 11.45 (s, 1H, NH), 10.35 (s, 1H, OH), 8.33 (s, 1H, N=CH), 7.99 (s, 1H, NH), 7.61 (d, *J* = 2.7 Hz, 1H, Ar-H), 7.25 (dd, *J* = 8.6, 2.7 Hz, 1H, Ar-H), 6.92 (d, *J* = 8.7 Hz, 1H, Ar-H), 3.26 (d, *J* = 4.4 Hz, 1H), 1.77 (dd, *J* = 11.8, 7.0 Hz, 2H), 1.66 (s, 3H), 1.61 – 1.46 (m, 2H), 1.46 – 1.27 (m, 1H), 1.27 (s, 4H), 1.23 (s, 0H), 1.11 (d, *J* = 9.9 Hz, 1H), 1.01 (s, 3H). ^{13}C NMR (101 MHz, DMSO-d_6) δ 175.10(C=S), 155.23, 136.10(C=N), 130.30, 124.19, 123.18, 122.22, 117.93, 65.27, 49.63, 46.94, 45.26, 34.25, 25.61, 22.90, 22.86, 22.82, 17.55 (Figure S9).

2-hydroxy-5-methylbenzaldehyde-4-(2′-isocamphanyl) thiosemicarbazone **(TSC-6)**. White solid; Yield 88.15%; ^{1}H NMR (400 MHz, DMSO-d_6) δ 11.32 (s, 1H, NH), 9.76 (s, 1H, OH), 8.34 (s, 1H, N=CH), 7.99 (s, 1H, NH), 7.41 (d, *J* = 2.2 Hz, 1H, Ar-H), 7.02 (dd, *J* = 8.3, 2.2 Hz, 1H, Ar-H), 6.77 (d, *J* = 8.3 Hz, 1H, Ar-H), 3.24 (s, 1H), 2.18 (s, 3H), 1.83 – 1.70 (m, 2H), 1.65 (s, 3H), 1.60 – 1.44 (m, 2H), 1.37 (tq, *J* = 12.1, 4.1 Hz, 1H), 1.25 (s, 4H), 1.09 (d, *J* = 9.9 Hz, 1H), 1.00 (s, 3H). ^{13}C NMR (101 MHz, DMSO-d_6) δ 175.04(C=S), 154.40, 138.03(C=N), 131.70, 127.74, 125.29, 119.98, 116.11, 65.19, 49.64, 47.04, 45.26, 34.25, 25.66, 23.12 – 22.73 (m), 20.14, 17.66 (Figure S10).

Part 3 Cartesian coordinates of the investigated compounds

xyz file giving the Cartesian coordinates of the investigated compounds

TSC-1

Atom	X	Y	Z
C(1)	-3.388	-2.612	0.213
C(2)	-2.996	-1.751	1.231
C(3)	-3.719	-2.114	-1.051
C(4)	-3.637	-0.747	-1.289
C(5)	-3.215	0.137	-0.285
C(6)	-2.922	-0.374	0.993
C(7)	-3.061	1.585	-0.583
N(8)	-1.972	2.255	-0.494
S(9)	0.598	3.847	-0.329
N(10)	-0.858	1.597	-0.117
C(11)	0.376	2.193	-0.107
N(12)	1.427	1.381	0.1
C(13)	0.842	-0.327	1.731
C(14)	2.079	-0.727	-1.855
C(15)	4.05	0.635	0.183
C(16)	3.484	-1.295	1.591
C(17)	3.057	-0.525	0.338
C(18)	1.508	-0.072	0.375
C(19)	3.116	-1.403	-0.949
C(20)	0.937	-0.87	-0.84
C(21)	0.952	-2.386	-0.559
C(22)	2.448	-2.767	-0.723
O(23)	-2.545	0.419	2.023
H(24)	-3.444	-3.684	0.411
H(25)	-2.753	-2.119	2.23
H(26)	-4.037	-2.791	-1.845
H(27)	-3.882	-0.341	-2.273
H(28)	-3.929	2.145	-0.946
H(29)	-0.907	0.584	-0.024
H(30)	2.293	1.906	0.147
H(31)	1.385	0.225	2.511
H(32)	-0.199	0.024	1.755
H(33)	0.839	-1.386	2.007
H(34)	2.308	0.318	-2.104
H(35)	1.893	-1.286	-2.784
H(36)	5.071	0.226	0.19
H(37)	3.927	1.186	-0.761

H(38)	3.985	1.348	1.02
H(39)	3.553	-0.624	2.46
H(40)	2.807	-2.116	1.856
H(41)	4.483	-1.727	1.425
H(42)	4.139	-1.471	-1.344
H(43)	-0.041	-0.526	-1.199
H(44)	0.327	-2.899	-1.304
H(45)	0.546	-2.642	0.428
H(46)	2.855	-3.319	0.133
H(47)	2.595	-3.396	-1.613
H(48)	-2.662	1.358	1.81

TSC-2

Atom	X	Y	Z
C(1)	-3.135	0.799	0.048
C(2)	-2.281	1.192	1.104
C(3)	-2.916	1.403	-1.222
C(4)	-1.901	2.328	-1.397
C(5)	-1.033	2.699	-0.359
C(6)	-1.269	2.127	0.905
C(7)	0.128	3.587	-0.61
N(8)	1.359	3.255	-0.458
S(9)	4.263	2.363	-0.163
N(10)	1.603	1.987	-0.064
C(11)	2.857	1.447	-0.016
N(12)	2.918	0.115	0.167
C(13)	1.133	-0.597	1.663
C(14)	1.857	-1.685	-1.922
C(15)	4.027	-2.381	0.239
C(16)	2.101	-3.232	1.501
C(17)	2.494	-2.369	0.297
C(18)	1.845	-0.892	0.338
C(19)	1.94	-2.939	-1.043
C(20)	0.95	-0.926	-0.944
C(21)	-0.222	-1.91	-0.775
C(22)	0.452	-3.301	-0.923
O(23)	-0.508	2.433	1.985
N(24)	-4.149	-0.1	0.251
C(25)	-4.966	-0.565	-0.861
C(26)	-4.319	-0.749	1.543
C(27)	-3.324	-1.87	1.832
C(28)	-4.285	-1.595	-1.757

H(29)	-2.389	0.796	2.111
H(30)	-3.537	1.143	-2.077
H(31)	-1.749	2.766	-2.387
H(32)	-0.037	4.6	-0.993
H(33)	0.806	1.355	-0.012
H(34)	3.875	-0.208	0.246
H(35)	1.847	-0.713	2.49
H(36)	0.751	0.431	1.716
H(37)	0.291	-1.273	1.848
H(38)	2.822	-1.188	-2.093
H(39)	1.371	-1.872	-2.891
H(40)	4.371	-3.425	0.243
H(41)	4.429	-1.909	-0.67
H(42)	4.477	-1.892	1.119
H(43)	2.609	-2.889	2.414
H(44)	1.023	-3.242	1.702
H(45)	2.416	-4.271	1.316
H(46)	2.566	-3.76	-1.421
H(47)	0.616	0.057	-1.299
H(48)	-0.958	-1.739	-1.574
H(49)	-0.754	-1.783	0.178
H(50)	0.233	-3.984	-0.093
H(51)	0.122	-3.798	-1.847
H(52)	0.073	3.188	1.803
H(53)	-5.879	-0.997	-0.431
H(54)	-5.292	0.3	-1.459
H(55)	-4.271	0.01	2.339
H(56)	-5.34	-1.151	1.569
H(57)	-3.533	-2.312	2.817
H(58)	-3.397	-2.666	1.077
H(59)	-2.289	-1.498	1.843
H(60)	-4.963	-1.896	-2.57
H(61)	-3.37	-1.189	-2.212
H(62)	-4.014	-2.495	-1.187

TSC-3

Atom	X	Y	Z
C(1)	-3.349	-0.63	0.165
C(2)	-2.762	0.032	1.25
C(3)	-3.437	0.001	-1.084
C(4)	-2.889	1.274	-1.233
C(5)	-2.259	1.938	-0.177

C(6)	-2.228	1.305	1.085
C(7)	-1.493	3.191	-0.417
N(8)	-0.212	3.258	-0.385
S(9)	2.837	3.24	-0.592
N(10)	0.446	2.116	-0.098
C(11)	1.797	1.967	-0.23
N(12)	2.265	0.715	-0.057
C(13)	1.082	-0.301	1.814
C(14)	1.417	-1.532	-1.782
C(15)	4.067	-1.333	-0.096
C(16)	2.757	-2.554	1.584
C(17)	2.64	-1.754	0.283
C(18)	1.592	-0.53	0.386
C(19)	2.051	-2.603	-0.884
C(20)	0.523	-0.972	-0.667
C(21)	-0.227	-2.236	-0.198
C(22)	0.801	-3.374	-0.436
O(23)	-1.641	1.878	2.16
O(24)	-3.794	-1.878	0.409
C(25)	-4.404	-2.596	-0.646
H(26)	-2.715	-0.456	2.225
H(27)	-3.902	-0.489	-1.937
H(28)	-2.921	1.757	-2.212
H(29)	-2.01	4.118	-0.687
H(30)	-0.111	1.276	0.036
H(31)	3.275	0.688	-0.132
H(32)	1.935	-0.082	2.472
H(33)	0.394	0.552	1.88
H(34)	0.561	-1.175	2.219
H(35)	2.129	-0.794	-2.176
H(36)	0.839	-1.959	-2.615
H(37)	4.702	-2.231	-0.122
H(38)	4.127	-0.861	-1.088
H(39)	4.508	-0.651	0.649
H(40)	3.285	-1.976	2.356
H(41)	1.79	-2.861	1.998
H(42)	3.343	-3.467	1.393
H(43)	2.819	-3.236	-1.35
H(44)	-0.171	-0.185	-0.992
H(45)	-1.126	-2.375	-0.818
H(46)	-0.571	-2.171	0.843
H(47)	0.967	-4.005	0.445
H(48)	0.47	-4.034	-1.251
H(49)	-1.398	2.8	1.98

H(50)	-4.682	-3.573	-0.236
H(51)	-5.31	-2.082	-1.004
H(52)	-3.708	-2.739	-1.487

TSC-4

Atom	X	Y	Z
C(1)	-3.184	0.308	-0.059
C(2)	-2.473	0.745	1.052
C(3)	-3.065	0.929	-1.303
C(4)	-2.182	1.997	-1.425
C(5)	-1.422	2.446	-0.338
C(6)	-1.598	1.828	0.914
C(7)	-0.377	3.489	-0.526
N(8)	0.881	3.285	-0.389
S(9)	3.87	2.727	-0.216
N(10)	1.274	2.04	-0.056
C(11)	2.587	1.652	-0.044
N(12)	2.804	0.334	0.118
C(13)	1.218	-0.549	1.742
C(14)	1.813	-1.624	-1.87
C(15)	4.204	-2.003	0.128
C(16)	2.497	-3.05	1.547
C(17)	2.689	-2.176	0.304
C(18)	1.869	-0.788	0.375
C(19)	2.114	-2.838	-0.983
C(20)	0.894	-0.959	-0.836
C(21)	-0.133	-2.076	-0.563
C(22)	0.695	-3.377	-0.747
O(23)	-0.918	2.209	2.014
Br(24)	-4.348	-1.176	0.128
H(25)	-2.577	0.272	2.029
H(26)	-3.638	0.58	-2.162
H(27)	-2.055	2.483	-2.394
H(28)	-0.667	4.496	-0.841
H(29)	0.565	1.313	-0.005
H(30)	3.795	0.126	0.165
H(31)	1.998	-0.531	2.516
H(32)	0.695	0.416	1.792
H(33)	0.498	-1.329	2.008
H(34)	2.694	-1.016	-2.119
H(35)	1.284	-1.89	-2.797
H(36)	4.672	-2.998	0.107

H(37)	4.473	-1.496	-0.811
H(38)	4.657	-1.453	0.968
H(39)	3.016	-2.618	2.416
H(40)	1.446	-3.196	1.824
H(41)	2.933	-4.044	1.359
H(42)	2.807	-3.584	-1.397
H(43)	0.412	-0.036	-1.183
H(44)	-0.943	-2.013	-1.304
H(45)	-0.602	-1.996	0.427
H(46)	0.624	-4.065	0.105
H(47)	0.362	-3.928	-1.637
H(48)	-0.437	3.04	1.868

TSC-5

Atom	X	Y	Z
C(1)	-3.208	-1.195	1.063
C(2)	-2.632	-0.258	1.91
C(3)	-3.459	-0.855	-0.267
C(4)	-3.137	0.406	-0.755
C(5)	-2.537	1.348	0.09
C(6)	-2.298	1.015	1.436
C(7)	-2.049	2.642	-0.464
N(8)	-0.809	2.939	-0.596
S(9)	2.163	3.509	-1.022
N(10)	0.085	2.011	-0.205
C(11)	1.429	2.113	-0.439
N(12)	2.147	1.007	-0.168
C(13)	1.263	-0.077	1.825
C(14)	1.663	-1.452	-1.714
C(15)	4.299	-0.67	-0.173
C(16)	3.3	-2.011	1.626
C(17)	2.991	-1.323	0.294
C(18)	1.74	-0.307	0.386
C(19)	2.523	-2.332	-0.799
C(20)	0.728	-1.001	-0.583
C(21)	0.251	-2.351	-0.008
C(22)	1.46	-3.293	-0.25
O(23)	-1.704	1.866	2.3
Cl(24)	-4.16	-2.039	-1.338
H(25)	-3.461	-2.191	1.43
H(26)	-2.429	-0.498	2.955
H(27)	-3.319	0.657	-1.802

H(28)	-2.764	3.39	-0.822
H(29)	-0.273	1.112	0.106
H(30)	3.138	1.164	-0.31
H(31)	2.095	0.322	2.423
H(32)	0.448	0.657	1.887
H(33)	0.915	-0.998	2.306
H(34)	2.204	-0.62	-2.184
H(35)	1.139	-2.027	-2.492
H(36)	5.091	-1.433	-0.172
H(37)	4.238	-0.266	-1.194
H(38)	4.626	0.132	0.508
H(39)	3.748	-1.305	2.34
H(40)	2.42	-2.457	2.104
H(41)	4.03	-2.817	1.454
H(42)	3.377	-2.838	-1.272
H(43)	-0.113	-0.376	-0.908
H(44)	-0.638	-2.688	-0.562
H(45)	-0.04	-2.287	1.05
H(46)	1.778	-3.836	0.648
H(47)	1.22	-4.045	-1.016
H(48)	-1.628	2.76	1.931

TSC-6

Atom	X	Y	Z
C(1)	-3.543	-1.799	0.74
C(2)	-2.94	-0.939	1.647
C(3)	-3.881	-1.378	-0.558
C(4)	-3.572	-0.066	-0.914
C(5)	-2.934	0.815	-0.026
C(6)	-2.644	0.377	1.277
C(7)	-2.55	2.176	-0.481
N(8)	-1.356	2.642	-0.519
S(9)	1.463	3.733	-0.696
N(10)	-0.351	1.832	-0.132
C(11)	0.965	2.181	-0.273
N(12)	1.866	1.208	-0.048
C(13)	1.12	-0.205	1.787
C(14)	1.95	-1.148	-1.815
C(15)	4.304	-0.013	-0.061
C(16)	3.514	-1.674	1.567
C(17)	3.132	-0.945	0.275
C(18)	1.702	-0.203	0.37

C(19)	2.92	-1.926	-0.918
C(20)	0.892	-0.983	-0.715
C(21)	0.656	-2.443	-0.281
C(22)	2.037	-3.114	-0.511
O(23)	-2.06	1.175	2.204
C(24)	-4.548	-2.322	-1.522
H(25)	-3.769	-2.824	1.045
H(26)	-2.698	-1.262	2.661
H(27)	-3.81	0.292	-1.919
H(28)	-3.328	2.847	-0.859
H(29)	-0.575	0.865	0.1
H(30)	2.814	1.56	-0.121
H(31)	1.819	0.304	2.466
H(32)	0.165	0.333	1.849
H(33)	0.954	-1.217	2.173
H(34)	2.342	-0.191	-2.185
H(35)	1.585	-1.741	-2.667
H(36)	5.231	-0.605	-0.074
H(37)	4.207	0.463	-1.048
H(38)	4.436	0.773	0.699
H(39)	3.775	-0.958	2.36
H(40)	2.721	-2.326	1.953
H(41)	4.399	-2.3	1.379
H(42)	3.876	-2.22	-1.373
H(43)	-0.034	-0.492	-1.039
H(44)	-0.116	-2.892	-0.921
H(45)	0.301	-2.528	0.755
H(46)	2.41	-3.66	0.365
H(47)	1.987	-3.832	-1.342
H(48)	-2.048	2.102	1.919
H(49)	-4.738	-1.835	-2.487
H(50)	-3.921	-3.209	-1.702
H(51)	-5.509	-2.679	-1.122

1. Goujot, D.; Cuvelier, M.-E.; Soto, P.; Courtois, F. A stoichio-kinetic model for a DPPH· -ferulic acid reaction. *Talanta* **2019**, *196*, 284-292,
